# Next-generation health monitoring: The role of nanomaterials in 3D-printed wearable devices

**DOI:** 10.1016/j.mattod.2025.03.005

**Published:** 2025-05-16

**Authors:** Chuchu Chen, Yonghao Fu, Yun Liu, Prashanta Dutta, Yuehe Lin, Dan Du, Kaiyan Qiu

**Affiliations:** a School of Mechanical and Materials Engineering, Washington State University, Pullman, WA 99164, USA; b Research School of Chemistry, Australian National University, Canberra, ACT 2601, Australia; c Department of Pharmaceutical Sciences, College of Pharmacy and Pharmaceutical Sciences, Washington State University, Spokane, WA 99202, USA; d Department of Translational Medicine & Physiology, Elson S. Floyd College of Medicine, Washington State University, Spokane, WA 99202, USA

**Keywords:** Nanomaterials, 3D printing, Wearable devices, Health monitoring

## Abstract

Wearable devices have made transformative advancements driven by the integration of nanomaterials, enhancing their versatility, sensitivity, and overall performance. The emerging 3D printing techniques revolutionize traditional fabrication, enabling the high-efficiency fabrication for sophisticated and miniaturized healthcare monitoring systems. This review summarizes the essential properties of nanomaterials and their roles in 3D printing and examines the pros and cons of various 3D printing methods. Key applications of 3D-printed wearable devices, showcasing the synergistic contributions of nanomaterials, are introduced to provide a comprehensive overview of the state-of-the-art progress and the promising prospects for next-generation healthcare monitoring.

## Introduction

Smart and wearable monitoring devices have revolutionized the landscape of healthcare by enabling fitness tracking, early diagnosis, and real-time assessment of disease states [1]. These wearable monitoring devices are designed to continuously, non-invasively, and in real-time monitor individuals’ physiological and biochemical parameters, offering innovative solutions for personalized health management [2–6]. Therefore, wearable monitoring devices play an important role in health management, highlighting the necessity for further study.

Nanomaterials play a pivotal role in the advancement of wearable devices by offering unique properties due to high surface-to-volume ratio, leading to an exponential increase in reactivity at the molecular level [7–12]. Nanomaterials are conventionally defined as materials with at least a dimension between 1 and 100 nm [13].

The discovery and utilization of nanomaterials have facilitated the emergence of numerous new technological domains in healthcare, such as nanomedicine, biosensors, and bioelectronics [14–17]. Previous studies highlight two main purposes for the application of nanoparticles in *in vitro* diagnostics: (1) enhancing the capture of trace amounts of targets and (2) amplifying the signal generated upon biomarker recognition [18–21]. Various nanomaterials have been used to improve the sensitivity and selectivity of wearable sensors [19,22]. For example, due to the large surface area, nanomaterials can increase support loading capacity and transport of reactants, resulting in a synergistic effect on signal amplification [23]. Additionally, metal organic frameworks (MOFs) are highly ordered nanomaterials that can improve the stability and selectivity of enzymes employed in sensors, making the sensors more selective for biomarkers [24–26]. Electrochemical biosensors based on nanomaterials have shown excellent sensitivity in aflatoxin detection in the field of food safety, with the limit of detection (LOD) generally reaching ng/ml [27]. Some nanomaterials, such as MnO_2_ nanosheets and cerium-doped Bi_2_MoO_6_ can even achieve pg/ml level detection [28,29]. A cobalt-functionalized TiO_2_ nanotubes-based electrochemical sensor, for severe acute respiratory syndrome coronavirus 2 (SARS-CoV-2), the virus responsible for coronavirus disease (COVID-19), enables rapid detection in approximately 30 s [30].

The evolving demand for more efficient wearable platforms has driven the need for biosensors with enhanced properties, such as high stretchability, high flexibility, light weight and fast response [31–33]. However, traditional sensors are limited by conventional fabrication techniques due to insufficient manufacturing scalability, poor durability, high cost of mass production, material waste and design constraints [34,35]. 3D printing, also known as additive manufacturing, has emerged as a transformative technology that enables the rapid and cost-effective fabrication of 3D functional devices by using a variety of precursor materials [36,37]. Unlike traditional manufacturing methods, 3D printing technology employs a simple one-step digitally controlled process to add the desired material layer by layer for complicated geometries and optimized functionality [38,39]. The development of versatile inks and advancement of 3D printing approaches allow to manufacture various wearable monitoring devices rapidly and cost-effectively [40]. In recent years, 3D printing has also been widely used in the manufacturing of wearable biosensors and has greatly promoted the development of wearable sensors. For example, soft and flexible biosensors made by 3D printing such as material jetting promote the development of wearable monitoring devices [41–43]. Similarly, material extrusion methods enable the integration of multiple sensing modalities into a single sensor, reducing the footprint of the device and enables miniaturization [43,44]. Notably, the high precision, flexibility, and cost-effective manufacturing of wearable devices through 3D printing offer unparalleled advantages, such as high resolution for intricated designs, unmatched freedom for tailored customization, reduced raw material waste, and scalability for mass production [38,45–47].

**FIG. 1:**
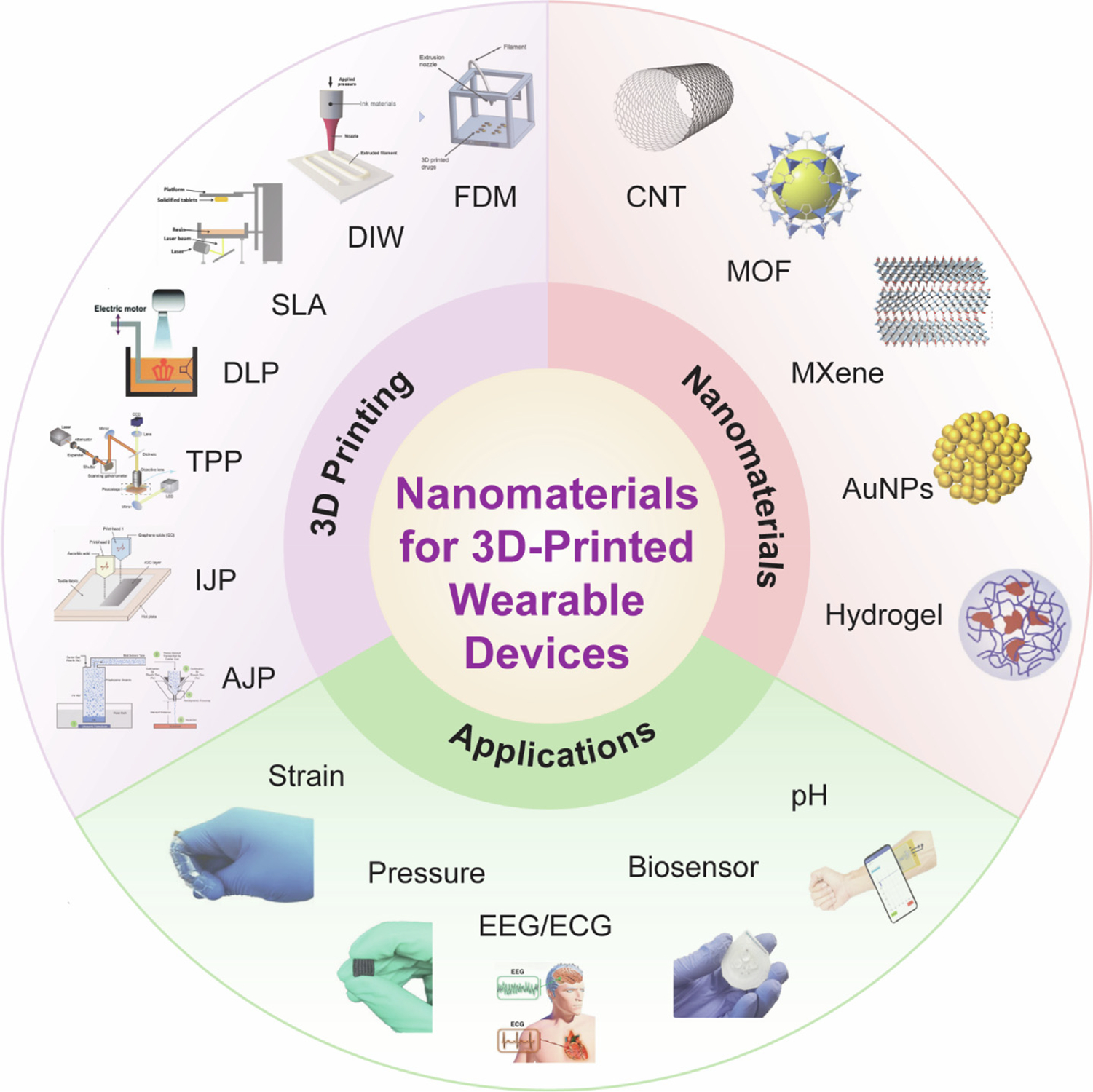
3D-printed wearable monitoring devices with different categories of devices (strain sensor, pressure sensor, electroencephalography (EEG)/electrocardiography (ECG) sensor, biosensor, and pH sensor), 3D printing approaches (fused deposition modeling-FDM, direct ink writing-DIW, inkjet printing-IJP, aerosol jet printing-AJP, stereolithography-SLA, digital light processing-DLP and Two-photon polymerization-TPP) and nanomaterials (carbon nanotube-CNT, metal–organic framework-MOF, MXene, gold nanoparticles-AuNPs, and hydrogel). Reproduced with permission from Refs. [43,52–68].

Fig. 1 shows wearable monitoring devices and the involved 3D printing techniques and nanomaterials for the devices.

To date, several reviews have highlighted the applications of nanomaterials for 3D printing, while others have summarized the advances, challenges, and prospects of wearable sensor devices [48–51]. However, to the best of our knowledge, no review comprehensively and systematically investigates the role of nanomaterials in 3D-printed wearable sensors.

This review aims to fill this gap by beginning with an in-depth exploration of nanomaterials, detailing their various functions in diverse 3D-printed wearable devices, and providing novel insights into the integration of nanomaterials with 3D printing technologies. First, we introduce commonly used nanomaterials for wearable devices, including carbon nanomaterials, metal nanomaterials, metal–organic frameworks, and biomaterials, highlighting their characteristics and roles in 3D-printed wearable devices. This is followed by a discussion of 3D printing methods and the applications of nanomaterials in various 3D-printed wearable devices. Finally, we address the challenges and future perspectives for wearable health monitoring devices, offering insights into next-generation wearable devices for health monitoring.

## Nanomaterials for 3D-printed wearable devices

Nanomaterials, including carbon nanomaterials, metal nanomaterials, MOFs, biomaterials, and other related nanomaterials, can be used in 3D-printed wearable devices for biosensing and health monitoring. Among these, the most widely used are Au, Ag, Cu, and Pt nanoparticles in metal nanoparticles; graphene, carbon black (CB), and carbon nanotubes (CNTs) in carbon nanomaterials; Fe-MOF, Zn-MOF, and Ni-MOF in MOFs; and hydrogel biomaterials with various functions.

### Carbon nanomaterials

**FIG. 2:**
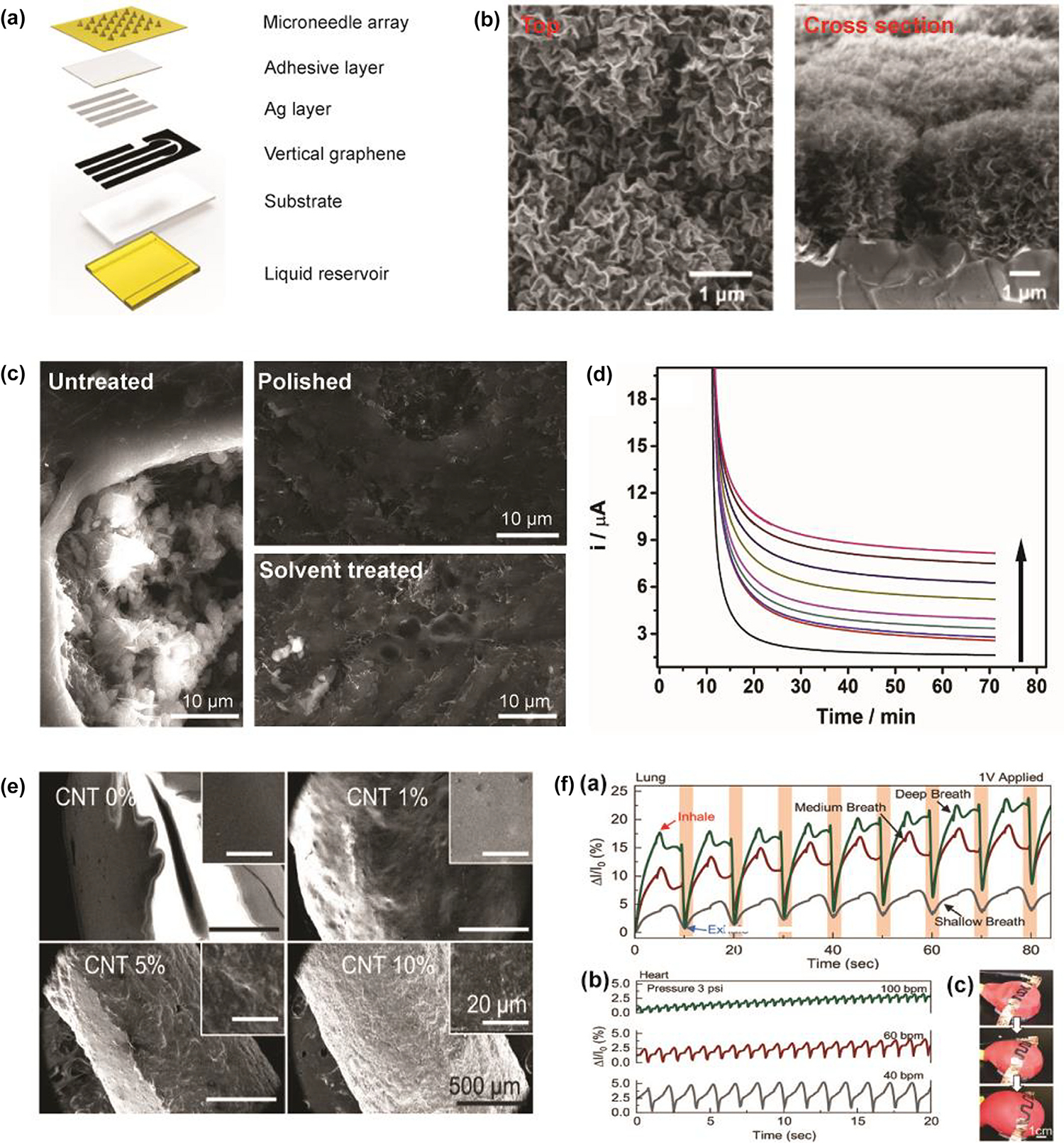
3D-printed wearable monitoring devices based on carbon nanomaterials. (A) Schematic diagram of the wearable microneedle sensor. The sensor consists of a microneedle array, adhesive layer, Ag layer, vertical graphene, substrate, and liquid chamber. (B) The top (left) and cross section (right) view SEM images of vertical graphene. Reproduced with permission from Ref. [75]. Copyright 2022 American Chemical Society. (C) SEM images of 3D-printed graphene-PLA surfaces: untreated (left), polished (top right) and solvent treated after polishing (bottom right). (D) Amperometric curves of samples containing various concentrations of glucose (Concentration increases in the direction of the arrow.). Reproduced with permission from Ref. [88]. Copyright 2019 Elsevier. (E) SEM images of CNT with different concentrations (0%, 1%, 5% and 10%). (F) Health monitoring applications of 3D-printed CNT-silicone structures. (a) Lung contractions mimicked by pumping air in and out of the red balloon. (b) Heart contractions mimicked with input and air release from the red balloon. (c) The red balloon mimicking lung and heart contractions. A pattern was printed onto a red latex balloon, and electrodes were attached to each end. Reproduced with permission from Ref. [92]. Copyright 2022 Wiley-VCH GmbH.

Carbon nanomaterials, which have superior properties such as high conductivity, thermal conductivity, chemical stability, and excellent mechanical properties, are widely used in various devices [69–72]. Among these carbon nanomaterials, research on the application of graphene, CB, CNTs, carbon nanofibers (CNFs), graphite sheet in 3D printing wearable monitoring devices has received widespread attention [73]. Table 1 lists several carbon-based materials that could be used in the fabrication of 3D-printed monitoring devices. With outstanding electrical conductivity, carbon nanomaterials are often used to make electrodes in biosensors [74]. Using 3D printing, Wang et al. designed a wearable vertical graphene-based microneedle biosensor (Fig. 2A) to detect β-hydroxybutyrate (HB) and glucose for real-time ketogenic diet management. From the top and cross section view of scanning electron microscopy (SEM) images (Fig. 2B), the graphene nanosheets form numerous pores and network structures which are beneficial to ion exchange. Additionally, the vertical shape of the graphene sheets can minimize the kinetic losses, leading to an increase of speed and efficiency of ion exchange and current collection. Therefore, the vertical graphene achieves a high sensitivity of 234.18 μA mM^−1^ cm^−2^ and low LOD of 1.21 μM, exhibiting excellent electrochemical properties. Besides, the microneedle array fabricated by stereolithography (SLA) method showed outstanding mechanical properties not only ensured continuous sampling of ISF but also reduced the pain during the sampling process. The modification of graphene on electrodes significantly improves the sensitivity of the sensor and reduced the LOD of HB and glucose to 8.51 μM and 1.21 μM, respectively [75].

The 3D-printed electrode based on 3D printable conductive polymers-based carbon nanomaterials is a common 3D product applied in fundamental electrochemistry and its applications [76]. Among carbon nanomaterials, CB, graphene and CNTs are popular ones used as conductive filler materials in studies [77,78]. CB exhibits higher conductivity [79], high surface area and stability [80]. Graphene has higher thermal conductivity [81] and low resistivity [82] and the nucleation sites of CNTs enable the reinforcement for polymer matrix [83]. Polymer materials, including polylactic acid (PLA) [84], acrylonitrile butadiene styrene (ABS) [85], polyimide (PI) [86] and polyurethane [87], can be reinforced with carbon nanomaterials to fabricate 3D printable conductive thermoplastic filaments, such as PLA/graphene, PLA/CB, PLA/CNTs, and ABS/CB [76]. Cardoso et al. developed a novel graphene/PLA electrode by fused deposition modeling (FDM) for biosensors to detect glucose, uric acid and nitrite in biological fluids [88]. Crosslinking with glutaraldehyde, oxygenated groups from PLA improve enzyme immobilization, while the graphene (conductive filler material) with excellent electrical conductivity increases the electrochemical properties of biosensors to achieve highly accurate detection of biomarkers. However, the SEM images of untreated 3D-printed graphene/PLA surfaces show that the PLA matrix could overlap the carbon structures on the surface of electrodes, resulting in low electron transfer and high resistivity (Fig. 2C) [88]. Fig. 2C shows that polishing (top right) and solvent treatment (bottom right) can improve the exposure of graphene nanoribbons on electrodes and the availability of electron transfer, thereby enhancing the performance of 3D-printed electrodes. Fig. 2D shows current obtained at +0.4 V for increased concentrations of glucose using amperometry. The detection for glucose proved the treatment enabled the performance improvement for the 3D-printed graphene/PLA electrodes. The electrodes also showed excellent performance for detection of uric acid and nitrite [88].

There is a growing need for conductive, biocompatible 3D printable inks for wearable devices used to monitor heart health, blood pressure and glucose which are also suitable for the mutability and customizability of 3D printing ink [89–91]. A cheap room temperature single-component, conductive, flexible CNT-silicone 3D printing ink was developed by Joung et al to solve the problem of harmful solvents, accessibility limitations and premature curing due to prior mixing in synthesis of conductive and stretchable elastomer-based 3D printing inks. A low-toxic, bio-sourced route to CNT dispersion was achieved by using butyl acetate instead of traditional solvent isopropyl alcohol. The one-part humidity curing mechanism makes the ink easier to handle than the previous two-parts polydimethylsiloxane (PDMS)-based conducting inks. In addition, this novel ink also has the advantages of short preparation process, extended lifetime within the 3D printing syringe, room-temperature synthesis, printing and curing, 3D printing self-supporting structures, and printing capability on various polar and nonpolar surfaces [90]. Based on this method, a one-part, conductive, flexible and 3D-printed CNT-silicone composite was developed to fabricate wearable electronics for motion detection [92]. Fig. 2E shows the SEM images of varying concentrations of CNT [92]. The surface roughness correlated with increasing concentration, and the excellent dispersion of CNT was demonstrated in SEM images. The increase in CNT concentration enables higher electrical conductivity and sensitivity to stimuli, but also leads to a decrease in mechanical properties, such as compressibility and stretchability. Therefore, an intermediate concentration of 5 % was found to be optimal for 3D printing CNT-silicone ink which combines excellent mechanical properties and strong electrical conductivity. Fig. 2F shows health monitoring applications of 3D-printed CNT-silicone structures. [92]. As shown in Fig. 2F (c), the pattern was printed on a red balloon and the electrodes were attached to each end. The pumping of air in and out of the balloon simulated the contraction of the lungs and heart. The simulation results for the lungs and heart are shown in Fig. 2F(a) and (b), respectively. The results indicate that shallow, medium, and deep breaths corresponded to progressively more significant changes in current. The 3D-printed CNT-silicone composite has been proved to have capability to distinguish between a fast heart rate and a slow heart rate over dozens of cycles. Besides, hypertension, hypotension, and normal blood pressure were also mimicked, and the current results remarkably corresponded to the cardiac physiology of hypertension and hypotension.

Attributed to their excellent conductivity, chemical stability and mechanical properties, carbon nanomaterials are widely used in the development of 3D printing inks and the production of 3D-printed wearable devices. First, the outstanding electrical conductivity and low resistivity of carbon nanomaterials make them ideal for fabricating electrodes with excellent sensitivity in wearable devices. The sensitivity of electrochemical wearable devices is significantly improved after the modification of carbon nanomaterials. Second, the development of conductive and biocompatible 3D printable inks for wearable devices has become a new trend in utilizing carbon nanomaterials. These inks are made by integrating carbon nanomaterials with polymer, where the nanomaterials serve as conductive filler material, and the polymers act as matrix materials. The resulting 3D printable conductive polymers-based carbon nanomaterials inks combine excellent mechanical properties with strong electrical conductivity. This type of 3D printing ink not only meets the mechanical properties required by wearable devices but also ensures the sensitivity and accuracy of detection, due to the excellent conductivity of the carbon nanomaterials.

### Metal nanomaterials

Research shows that metal and metal oxide nanoparticle films such as gold (Au), platinum (Pt), silver (Ag), palladium (Pd), copper (Cu) and zinc oxide (ZnO) can be deposited on sensor electrode materials, for improving the electrochemical performance of sensors and biosensor electrode devices [76]. At present, many studies have proven that metal nanomaterials have a good modification function for electrodes. For example, sensor electrodes modified with metal nanoparticles such as Au, Ag, Pt and Pd can enhance their electrocatalytic activity for hydrogen peroxide [95–97]. At the same time, metal conductor ink material is also an indispensable part of 3D-printed electrodes [98]. In other studies, researchers also integrated carbon materials into conductive inks [99], but compared to carbon materials, metal conductor ink materials have higher conductivity [100]. Table 2 shows metal nanomaterials used in 3D-printed wearable monitoring devices.

**FIG. 3:**
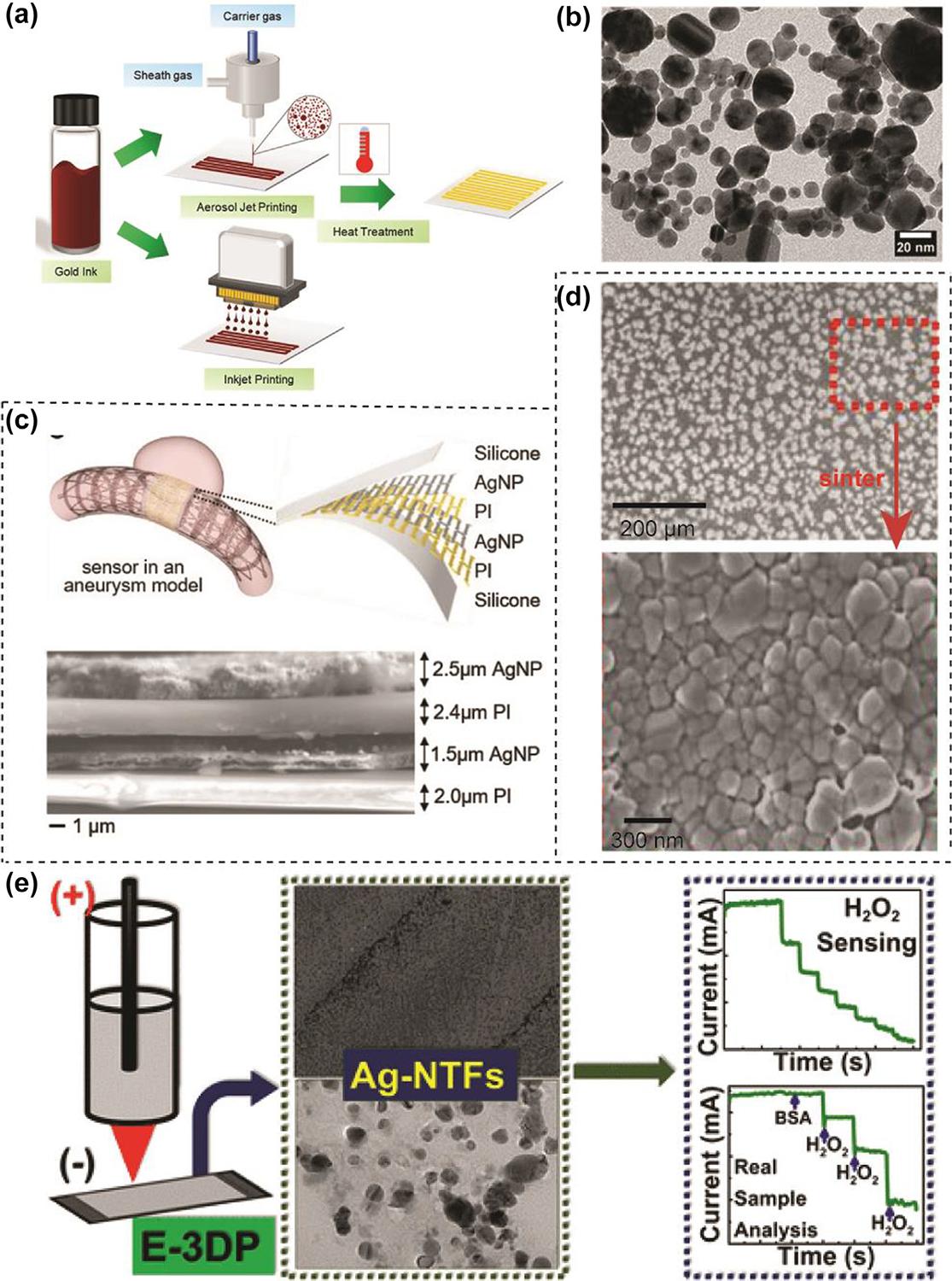
3D-printed wearable monitoring devices based on metal nanomaterials. (A) Schematic illustration of compatible aqueous Au nanomaterial inks for printed electronics. (B) TEM image of AuNPs in Au nanomaterial inks. Reproduced with permission from Ref. [107]. (C) Illustration of the AJP-enabled fabrication of an implantable flow sensor in an aneurysm model. (D) SEM images of AgNPs as printed (top) and after a sintering process (bottom). Reproduced with permission from Ref.[113]. (E) 3D-printed nanoscale-thick silver thin films for electrochemical sensing. Reproduced with permission from Ref. [114]. Copyright 2023 American Chemical Society.

Au nanomaterials possess properties in high electrical conductivity, mechanical flexibility, facile surface, functionalization, biocompatibility, wide electrochemical sensing window and extraordinary optical properties, making them potential materials for use in biosensors [101–104]. Due to the poor flexibility, bioactivity, stretchability and electrocatalytic activity of bulk gold, it is crucial to apply Au nanomaterials, such as gold nanoparticles (AuNPs) and gold nanowires (AuNWs) to 3D-printed biosensors [105]. Compared to Ag and Cu nanomaterials, Au has relatively lower electrical conductivity but better biosafety and biocompatibility [106]. Besides, Au materials are easier for surface functionalization, which is beneficial for their electrochemical sensing applications. Meanwhile, wearable sensors with the function of direct signal readout could intuitively and rapidly monitor human health information due to the localized surface plasmon resonance properties of Au nanomaterials. A multiple compatible Au ink for 3D printing and wearable electronics was developed in previous studies. Fig. 3A shows that the Au ink could be printed by aerosol jet printers and ink jet printers to fabricate Au electrodes. The spherical shape and size (6–50 nm) of AuNPs were observed in Fig. 3B. This low-cost and low-temperature sintering AuNPs ink is compatible with both aerosol jet printing (AJP) and inkjet printing (IJP) for highly conductive printed electronic applications [107]. Ye et al. developed a wearable aptamer nanobiosensor for non-invasive female hormone (oestradiol) monitoring in sweat. The AuNPs-MXene-based electrode provides extraordinary sensitivity and ultra-low LOD of 0.14 pM. The biorecognition interface and working electrode of this monitoring device were scalable inkjet printed by AuNPs (22 nm), which greatly improved the electrochemical active surface area for subsequent modifications. Meanwhile, MXene nanosheet was inkjet-printed on the surface of AuNPs to improve the electrical conductivity and the efficiency of charge transfer. The study shows that both AuNPs and MXene play important roles in device performance by comparing different modified materials on electrodes [108].

Ag nanomaterials have received widespread attention in the field of sensors due to their excellent catalytic activity, biocompatibility, low toxicity, stability, and reproducibility [109,110]. In the electrochemical sensor used to detect sulfamethoxazole, Oliveira et al. fixed the composite material modified with AgNPs on a 3D-printed ABS carrier using electrodeposition method and observed that the current signal response of the modified sensor increased by 30 % compared with the bare sensor [111]. Ali et al. fabricated a multi-length scale electrode architecture, which consisted of AJP micron-scale and mesoscale hollow Ag pillars. This electrode was coated with atomic-level thin reduced graphene oxide, achieving an improvement in the limitation of detection for dopamine from micromolar to femtomolar concentration [112]. Metal nanoparticles can also be combined with polymers to create 3D-printed wearable devices. A wireless monitoring of cerebral aneurysm hemodynamics with stretchable electronics was fabricated by AgNPs and PI via AJP. Fig. 3C shows the multilayered device structure and its components include silicone layers, AgNPs layers and PI layers. The SEM images show that sintering AgNPs can yield a high density of the printed AgNPs (Fig. 3D). The author also demonstrated extremely small size and mechanical properties of the sensor allow seamless integration with an expanded stent followed by compression and deployment without damage [113]. Singh et al. developed nanoscale-thick silver thin films (Ag-NTF) on indium tin oxide-coated glasses through an electrochemical 3D printing process. Electrochemical surface activity studies showed that the 3D-printed electrodes obtained using electrodeposition had excellent electrochemical activity and could be used for biosensing hydrogen peroxide (Fig. 3E). TEM images revealed that Ag-NTF particles on electrodes were spherical with an average size of 21 nm. Ag-NTF biosensor had a sensitivity of 25 ± 1 μA mM^−1^ cm^−2^ and a LOD of 1.0 μM. The excellent selectivity of biosensor was reflected in the ability to accurately measure glucose concentration in the presence of interferences [114].

Cu conductive inks with good high-temperature conductivity have attracted great interest due to their increasing contribution to the field of printed electronics [115]. Li et al. used 3D printing to fabricate high-temperature Cu electronic sensors for use in resistance thermometer sensors and flexible electronic applications [98]. Redondo et al. successfully prepared 3D-printed Cu electrodes by FDM method followed by a sintering step and evaluated its feasibility as the non-enzymatic sensing of glucose. Because of their good electrocatalytic properties, metal nanoparticles are often used in various electrochemical sensing applications. For example, creatinine can bind with various transition metal ions such as Ag(I), Zn (II) and Cu (II) [116,117], therefore, a reduced graphene oxide (rGO) stabilized binary Cu-iron oxide-based nanocomposite on 3D-printed Ag-electrode (Fe-Cu-rGO@Ag) was applied to measure level of blood creatinine. In biosensing process, Cu nanoparticles act as an excellent electron-transfer mediator through rGO and Fe atoms serve as active sites for creatinine oxidation. Modification of metal on 3D-printed Ag electrodes significantly improves sensor detection sensitivity and range [118]. Kumar et al. improved the sensor’s performance in detecting glucose and sucrose via coating Cu and nickel on the 3D-printed carbon electrodes surface [119].

Compared to carbon nanomaterials, metal nanomaterials exhibit better conductivity and lower resistivity. Besides, virous metal nanomaterials possess unique properties due to the diversity of metal types. Various properties, such as mechanical flexibility, facile surface modification, biocompatibility, low toxicity, catalytic activity, make metal nanomaterials widely used in wearable sensors. Moreover, metal nanomaterials inks are commonly used in 3D-printed electrodes for wearable devices or combined with carbon nanomaterials and polymers to use. The diverse and specialized properties of metal nanomaterials enable them to fulfill the specific requirements in the manufacturing of wearable devices. For example, Cu conductive ink with high-temperature conductivity is used in resistance thermometer sensors, while Au nanomaterial ink, with its excellent biocompatibility and ease of surface functionalization, is utilized to fabricate working electrodes in 3D-printed wearable devices.

### Metal-organic frameworks

The MOFs are new hybrid porous crystalline materials and constructed by alternatively connecting metal ions/clusters with organic linkers [130,131]. Compared with other porous materials, MOFs possess outstanding properties, including high surface area, tunable size of nanopores and uniformly structured cavities [131–133]. In current non-enzymatic electrochemical glucose biosensors, the MOF for directly catalyzing the oxidation of glucose, are utilized as electrode modifiers [134]. MOFs with specific catalytic properties, such as Co-MOF, Fe-MOF and Ni-MOF, can be used as artificial nanozymes to mimic and replace natural enzymes [135–137]. Thus, the application of MOFs in biosensing area overcomes the limitations of natural enzymes such as low stability and high cost [138,139]. Single atom catalysts (SACs) based on the structure of MOFs offer many advantages in wearable sensors, effectively helping to overcome the shortcomings of traditional materials [140,141]. Table 3 shows 3D-printed wearable monitoring devices based on MOFs.

The mechanism of MOFs in glucose sensing is as follows. First, the absorption of glucose occurs on the surface and/or inside the pores of the MOFs. Afterwards, the redox activity of metal sites of the MOFs causes the oxidation of glucose to gluconolactone. The sensing results can be obtained by scanning potential at the working electrode. Recently, a 3-electrode 3D-printed device was developed for the voltametric determination of glucose concentration in artificial sweat. The device was modified with a water-stable and non-toxic metal–organic framework of iron (Fe (II)-MOF) and it can detect glucose in acidic epidermal sweat environment without any interference from other co-existing metabolites [142]. In previous research, a highly efficient Ca-MOF, as an electrode modifier, was first combined with 3D printing to produce a novel fully integrated lab-in-a-syringe device for the sensitive determination of Hg (II) by anodic stripping voltammetry. This combination enables the fabrication of a simple, low-cost and sensitive electrochemical sensor for Hg (II), which is suitable for on-site applications [143]. Besides, a MOF and 3D printing were integrated for the synthesis of a tough MOF-based ionogel (MIG) for colorimetric and mechanical sensing. This method demonstrates a novel and facile strategy for the construction of multifunctional sensors based on MOF-based composite materials [144].

**FIG. 4:**
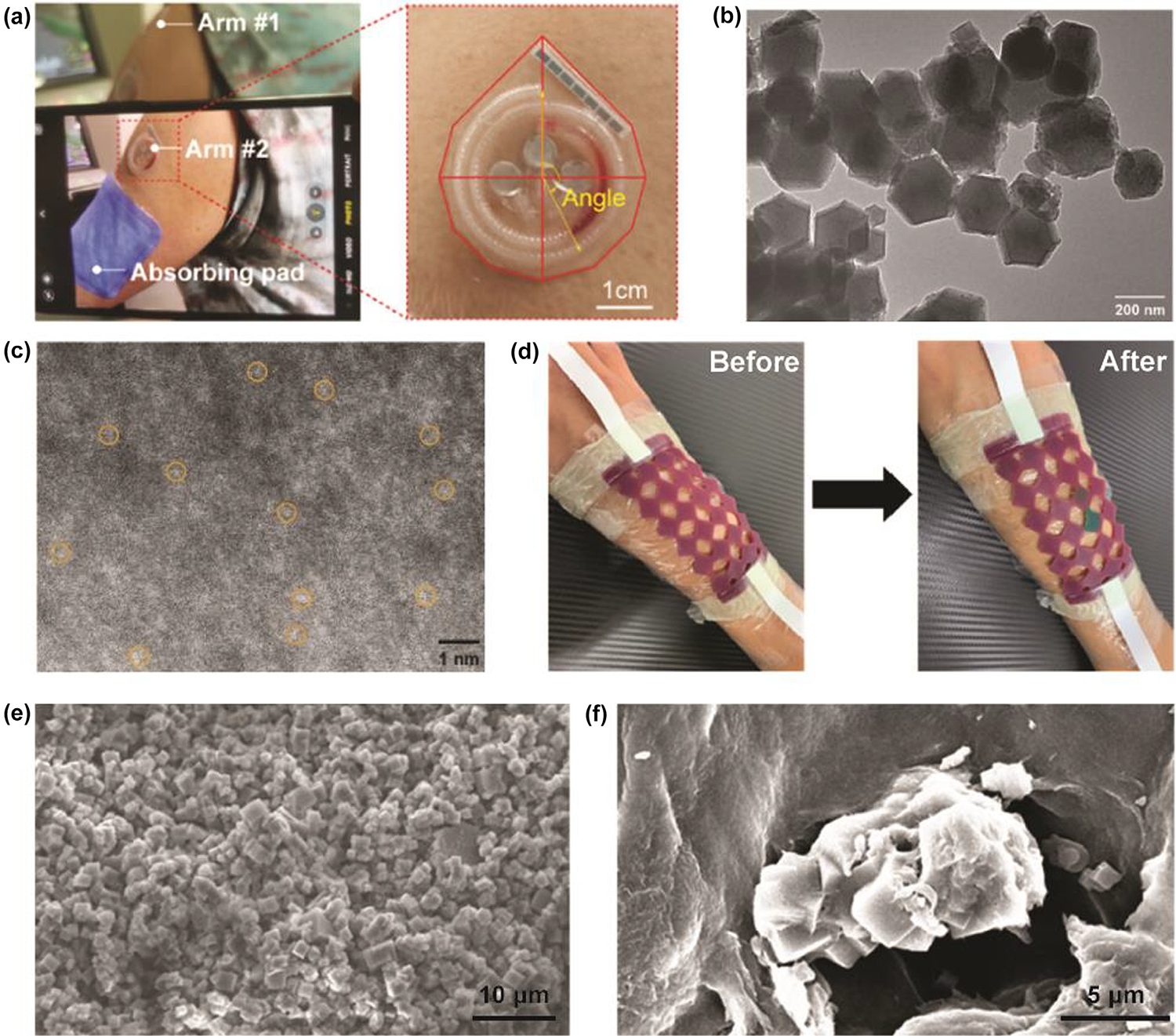
3D-printed wearable monitoring devices based on MOF. (A) Optical images of application of the biosensor using a smartphone. (B) TEM image of ZIF-8@Fe (NO)_3_. (C) HAADF-STEM image of ZIF-8@Fe(NO)_3._ Reproduced with permission from Ref [43]. Copyright 2024 American Chemical Society. (D) Photos of the sensor before and after the exposure of HCl solution. (E) SEM image of MOF-525 powder. (F) SEM image of MIGP with 0.2 wt% of MOF-525. Reproduced with permission from Ref [144]. Copyright 2022 American Chemical Society.

SACs with dispersed metal active sites offer excellent advantages in catalysis and selectivity for catalytic reactions, and SAC-based signal amplification strategies have great potential for application in biosensors [145,146]. Chen et al. designed a 3D-printed flexible microfluidic health monitor for *in situ* sweat analysis and biomarker detection. ZIF-8@Fe(NO_3_)_3_ with ultrasensitive peroxidase activity was developed and utilized in colorimetric bioassays to improve sensitivity and accuracy (Fig. 4A). As a Fe-N-C SAC, ZIF-8@Fe(NO_3_)_3_ owes to isolated iron active sites, mimicking the structure of a natural enzyme [147–149]. Fig. 4B shows TEM image of ZIF-8@Fe(NO_3_)_3_, in which similar size and shape (rhombic dodecahedral structure) were observed. In high-angle annular dark-field scanning transmission electron microscopy (HAADF-STEM) images, it can be observed that the above iron atoms active sites (bright spots) are dispersed in the MOF structure, which is also the reason why this material has extremely high peroxidase activity (Fig. 4C) [43]. Chen et al. also developed 3D-printed biocompatible hollow microneedle-based electrochemical sensor for wireless glucose monitoring based on ZIF-8@Fe(NO_3_)_3_. Due to the excellent peroxidase-like activity of ZIF-8@Fe(NO_3_)_3_, reduction of H_2_O_2_ resulting from glucose oxidation is enhanced dramatically on electrode surface, leading to a high response current at a low potential. In this way, ZIF-8@Fe (NO_3_)_3_ significantly improves the selectivity and sensitivity of electrochemical sensing devices [150].

Pal. et al. developed 4D printing of MIG to fabricate wearable sensors with colorimetric and mechanical responses (Fig. 4D). Fig. 4E and F shows the SEM images of MOF-525 and MIG which were used in wearable sensors. The SEM image of the MOF-525 powder reveals that the material is composed of cubic crystals with a size of 1–2 μm. The SEM image of MIG demonstrates the presence of MOF-525 on the polymer surface [144].

As a type of new hybrid porous crystalline material, MOFs possess a high surface area, tunable size of nanopores and uniformly structured cavities. More importantly, MOFs have excellent catalytic performance and enzyme-like activity, enabling them to serve as artificial nanozymes to replace natural enzymes. The low cost and high stability of MOFs address the limitations of natural enzymes. Their exceptional catalytic properties not only make MOFs widely used in 3D-printed wearable electrochemical devices but also suitable for wearable colorimetric sensors. In recent years, novel MOFs with diverse enzyme-like activities have been developed for wearable devices, which significantly improves the detection stability and further reduces costs when combined with 3D printing.

### Biomaterials

Because of the potential to be used in biosensing, tissue-engineered scaffolds and implants, electrically conductive biomaterials are gaining increasing interest [152]. Smart biomaterials have broad application prospects, ranging from tissue engineering, drug delivery and biosensors to more recently explored environmental applications [153]. Meanwhile, the appearance of 3D printing technology has opened up the more accurate and rapid automated biomanufacturing road. The incorporation of DIW with smart materials introduces freedom in design, stability and multifunctionality into the printed structures [54]. Compared to traditional materials, novel biomaterials offer advantages in adaptability, self-control, sustainability, and intelligence [154,155]. Table 4 shows biomaterials used in 3D-printed wearable monitoring devices.

Hydrogels are among the most frequently used biomaterials in tissue engineering. Although electrically conductive hydrogels have been comprehensively reviewed in the literature, the combination of electrically conductive hydrogels with 3D printing technology has only emerged recently [152]. The electrical conductivity of biomaterials can provide promising control over biomaterials function, such as drug release control, tissue repair and biosensing [156–158]. In addition, hydrogel not only offers advantages of easy recognition and natural degradation by the body, synthetic hydrogel but also is popular for 3D BP due to enhanced mechanical properties, biocompatibility and less batch-to-batch variation [159].

**FIG. 5:**
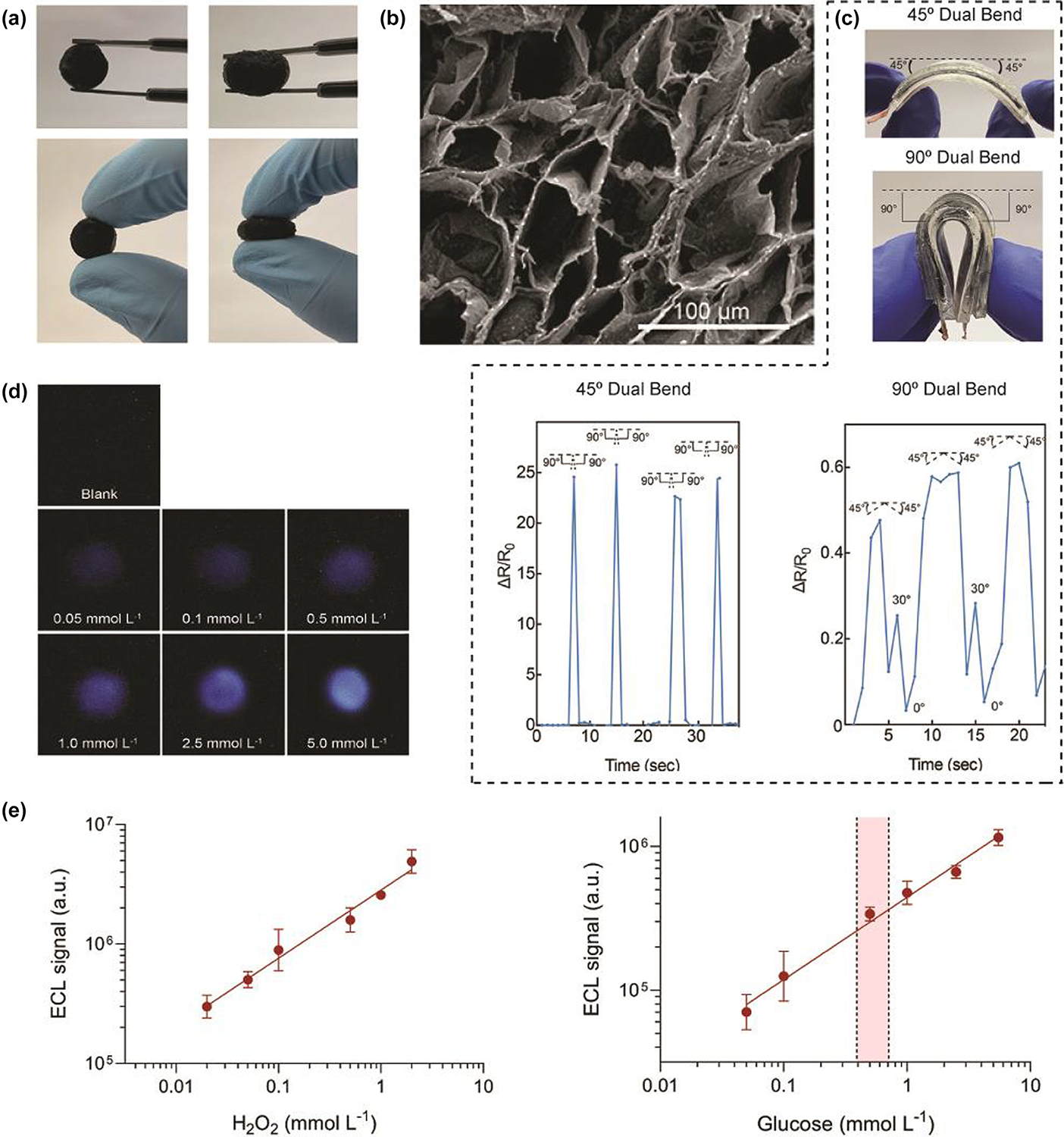
3D-printed wearable monitoring devices based on biomaterials. (A) Photographs of the nanoengineered composite hydrogels. (B) SEM images of a transverse cross-section of hydrogels. (C) The wearable hydrogel device was bent at 45° (left top) and 90° (left bottom) from both ends and the corresponding resistance change was recorded for each bending motion cycle. Reproduced with permission from Ref. [160]. Copyright 2022 American Chemical Society. (D) ECL images acquired using a smartphone for the detection of glucose standard solutions. The concentrations of glucose in standard solutions were 0 (blank), 0.05, 0.1, 0.5, 1.0, 2.5 and 5 mmol·L^−1^ respectively. (E) Calibration curve for the analysis of H_2_O_2_ (left) and glucose (right) obtained using the hydrogel-based ECL device. Reproduced with permission from Ref. [165].

The traditional method to synthesize materials for fabricating flexible electronics is combining conductive nanomaterials and polymeric hydrogels that are cross-linked using toxic photoinitiators. To avoid using the method involving toxic chemicals, as well as increasing the biocompatibility of materials, Deo et al. developed shear-thinning hydrogels which were designed through a facile vacancy-driven gelation of MoS_2_ nanoassemblies with naturally derived polymer-thiolated gelatin as biomaterial inks for 3D-printed flexible bioelectronics. Fig. 5A intuitively shows the elastomeric characteristics, flexibility, and robustness of the hydrogels. Besides, an interconnected hydrogel network was observed in SEM images of transverse cross-sections of hydrogels, which explains why hydrogels have such mechanical properties from a structural perspective (Fig. 5B). Meanwhile, when the wearable sensor made of this hydrogel was bent or twisted at different angles, it would produce specific resistance change characteristics, which indicates the elastomeric hydrogel to be sensitive under external strain (Fig. 5C). This also demonstrates that this specific hydrogel can be used in elastomeric flexible wearable bioelectronics to accurately capture human motion dynamics and patterns [160]. Application of 3D printing to develop hydrogel microchannels for wearable devices is also a recent emerging research area. Chen et al. developed the micromachining of microchannels in poly(HEMA) hydrogels using 3D printing technique for wearable contact lens biosensor [161]. The flow administration in the microchannel-containing poly (HEMA) contact lens showed the flow of liquid in the sensor and proves the feasibility of microchannels. The artificial tears with different pH values in the sample pools could spontaneously flow into the storing reservoirs, and then to the sensing area through the contact lens’ microchannels to detect the pH of samples. Additionally, the microchannel could also be applied to Na^+^ selective biosensor. The Na^+^ electrode adhered firmly onto the hydrogel substrate by using the hydrogel precursor as an adhesive promoter. Groll et al. defined bioinks as “a formulation of cells” suitable for processing by an automated bio-fabrication technology that may also contain biologically active components and biomaterials [162]. The even microcarriers of cells, cell aggregates and bioactive hydrogels are commonly used as bioinks in 3D bio-printing [163]. The hydrogels can be divided into natural and synthetic bioinks [164].

3D-printed sensors can also be combined with electrochemiluminescence (ECL) to take advantage of the fast response and high sensitivity of this detection method. A portable 3D printed micro-ECL biosensor based on the luminol/H_2_O_2_ ECL system was developed for the detection of glucose, with PLA doped with CB used as the electrode material. Fig. 5D shows the ECL images obtained by detecting glucose standard solutions with concentrations ranging from 0.05 to 5 mM. Fig. 5E also shows the calibration curve for analysis of H_2_O_2_. The ECL system was successfully used to sense H_2_O_2_ in 3D-printed sensor, and the luminol/H_2_O_2_ system could be coupled with glucose oxidase for glucose detection. The ECL biosensor showed excellent detection capability for glucose with a detection limit of 60 μmol L^−1^. The biosensor was also used for the detection of artificial serum samples and was suitable for point-of-care analysis [165].

Biomaterials with stability, self-control, sustainability, and intelligence have broad application prospects. The incorporation of DIW and biomaterials introduces design freedom of wearable devices. Undoubtedly, hydrogels are a crucial type of biomaterial used in 3D-printed wearable devices. In addition to their elastomeric characteristics, flexibility and robustness, the electrical conductivity of biomaterials opens up more possibilities for 3D-printed wearable devices. For example, 3D-printed strain sensor effectively combines mechanical properties with resistance change capabilities to detect external stress. Therefore, the continuous development of new biomaterials will inevitably bring more progress and possibilities to 3D-printed sensors.

### Other nanomaterials

In addition to the common nanomaterials mentioned above, other nanomaterials with various properties are also used for manufacturing of 3D printed monitoring devices. A 3D-printed biosensor consists of a microelectrode array coated with sintered Au nanoparticles, graphene and specific antibodies. It was manufactured by a high-resolution AJP 3D nanoprinter to detect acute respiratory syndrome coronavirus-2 (SARS-CoV-2) spike antigens. The application of 3D printing and introduction of nanomaterials make the biosensor have low limit-of-detection (9.2 fm), high analytical sensitivity (100 fm), rapid detection (43 s) and high selectivity [168]. Covalent organic frameworks (COF) are extended structures with periodic molecular orderings and inherent porosity and possess advantages of high stability and surface area, low density, and adjustable hole size [169]. Wang et al. first reported a magnetic COF and bovine serum albumin (BSA) (as the chiral surface) functionalized 3D-printed electrochemical chiral sensor, which opens up a new approach for enantiomer recognition [170]. The COF and Al_2_O_3_ nanoparticles were coated on 3D-printed electrodes constructed by FDM to modify and enhance their properties. For detecting biomarkers such as ascorbic acid, catechol and dopamine, the Al_2_O_3_/COF 3D-printed electrodes demonstrated high sensitivity [171].

## 3D printing technologies for wearable health monitoring devices

The unique properties of nanomaterials, as described in this section, significantly enhance the performance of wearable devices. Meanwhile, 3D printing techniques provide diverse and flexible fabrication methods, enabling the effective integration of these materials and expanding their potential applications in wearable health monitoring. 3D printing is a rapid layer-by-layer prototyping process that revolutionized the development of wearable health monitoring devices by fabricating 3D objects under computer-aided design (CAD) or computed tomography (CT) scan [172–174]. The fabrication process begins with conceptual 3D objects created by CAD modeling software and then converted into universal STL files [175]. After that, STL files are segmented by slicer software to generate G-code files that define the layer-by-layer construction details [176,177]. The early 3D printing technology called SLA was first proposed by Charles W. Hull in 1986 [178], which paved the way for the further development of additive manufacturing. Compared with traditional methods, 3D printing exhibits superiorities in terms of versatility for complicated 3D geometries, customizability, ease of accessibility, and relatively low cost [179,180]. According to the ISO/ASTM 52900:2015 standard, the American Society for Testing and Materials (ASTM) categorizes these 3D printing techniques into seven domain types based on the difference in working principle and raw materials: material extrusion, vat photopolymerization, material jetting, binder jetting, directed energy deposition, powder bed fusion, and sheet lamination [181–183].

**FIG. 6:**
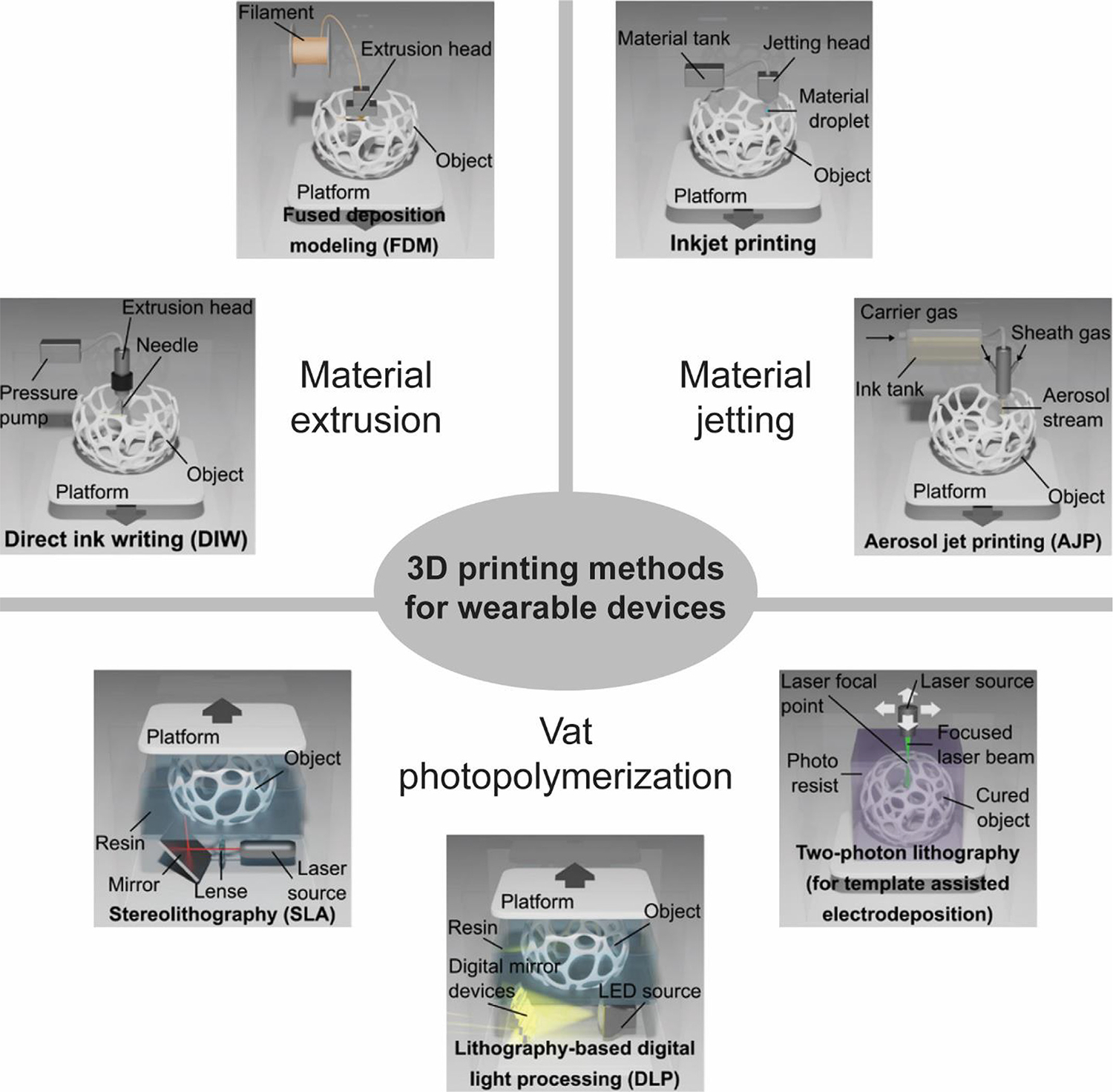
Schematics of 3D printing methods for wearable devices. Images adapted with permission from [185].

Benefitting from the advances in raw materials, various 3D printing technologies have been exploited to fabricate wearable devices, such as materials extrusion, vat photopolymerization, and material jetting [184], which are briefly elaborated in Fig. 6.

### Material extrusion

Material extrusion-based techniques, such as FDM and DIW, have been utilized to fabricate wearable devices [37,186].

Among extrusion-based methods, FDM, also named as fused filament fabrication (FFF), is a popular and extensively used technique because of its simplicity and low cost [187,188]. During the printing process, thermoplastic filaments are heated to a semi-molten state and then extruded onto the platform to solidify into final objects [173]. The layer thickness [189,190], filament infill pattern and density [191,192], build orientation [193], operation temperature [194], printing speed [195], and air gap [196] are vital factors affecting qualities and performances of the FDM processed parts, including dimensional accuracy, surface roughness and mechanical strength [197–199]. A spool of thermoplastic filaments including PLA [200,201], ABS [202], and thermoplastic polyurethane (TPU) [203], have been utilized for FDM fabrication of wearable devices. These composite materials for FDM processing are supposed to be in a feedstock filament form with sufficient melt viscosity [204]. However, the addition of nanocomposites into the filament leads to inhomogeneous dispersion and void formation challenges [205]. Moreover, the subsequent thorough removal of supporting materials remains difficult [206].

Direct ink writing, also known as robocasting, is a state-of-the-art pressure-driven extrusion technique that has been customized to print complex 3D geometries with printable viscoelastic inks [207]. Unlike FDM, DIW features unique advantages due to the versatile fluidic inks [54]. To date, various materials with tailored rheological properties, such as polymers, biomaterials, and hydrogels, have served as functional inks for the fabrication [208]. The printing parameters, such as extrusion pressure, printing speed, and nozzle size mainly determine the printing resolution and accuracy [209–211]. After deposition, the printed objects can be solidified by subsequent procedures, namely solvent evaporation, gelation, solvent-driven reactions, thermal treatment, and photocuring [212]. Nanomaterials, for example, nanoparticles, nanotubes, etc. have been added to functionalize the inks [213,214]. Despite lower resolution and precision, this technique is still highlighted for the future of wearable sensors [215,216], biomedical engineering [217–219], electronics [210], soft actuators and robotics [220–222].

### Vat photopolymerization

As suggested by the name, vat polymerization is characterized by the polymerization process of photopolymer resins via specific light sources. The most common methods of vat photopolymerization include SLA, DLP, and TPP [223].

SLA is a pioneer rapid prototyping technique with excellent resolution and superior accuracy [42,224]. During the printing process, liquid light-sensitive resins are exposed and photopolymerized using specific photoinitiators, such as ultraviolet (UV, 190–400 nm), visible light (400–700 nm), and infrared (700–1000 nm) [225]. After the radicalization, residual uncured resins are washed off and the obtained fabricated products are subjected to a post-curing or heating treatment to achieve the desired resolution and mechanical performances [226]. To polish the dimensional quality, several parameters, in particular, layer thickness, laser intensity, exposure time, and orientation should also be optimized [227–230]. Moreover, the type of resin is significantly influential on the mechanical properties of the final products, including strength, flexibility, durability, and biocompatibility [231,232]. To reinforce the functional performance of resins, nanomaterials have been introduced to develop polymer matrix composites [233–235]. Nevertheless, the following uneven dispersion and bubble retention issues that arise during the mixing process notably deteriorate the quality and impede its applications [236]. Therefore, post-processing is of great importance to address these challenges [16,237,238].

Similar to SLA, digital light processing is known for its high resolution and precision [239]. The major difference lies in the light source for photopolymerization [240]. The DLP method employs a projected light source together with a digital micromirror device (DMD) or liquid crystal display (LCD) screen to solidify the entire surface at once [241]. Whereas the SLA utilizes a laser beam to cure the resin path dot-by-dot [242,243]. Thus, DLP demonstrates faster speed and higher precision than SLA. This method can only print small models due to the limited exposure area [244]. So far, the incorporation of nanomaterials into photocurable resins for DLP printing expands its application in the field of tissue engineering [245,246] and medical applications [247].

Two-photon polymerization, also referred to as direct laser writing (DLW) or multi-photon lithography (MPP), is a newly emerged AM method over the past two decades that offers unparalleled resolution and precision for micro- and nanoscale fabrication [248]. TPP involves a nonlinear optical activation process using near-infrared (NIR) femtosecond lasers as light sources [249]. To date, researchers have doped carbon nanomaterials, metal nanowires, and magnetic nanoparticles into the photoresist to induce additional conductive properties [250–252]. Regardless of these limitations associated with its scalability, low speed, and expensive equipment, the considerable potential of this cutting-edge technology has been immensely explored for the manufacturing of photonics, drug delivery, tissue engineering, and microfluidic devices [253].

### Material jetting

The material jetting, including material jetting and aerosol jet printing, demonstrates prominent advantages for creating highly structured devices with high resolution and precision [254,255].

Inkjet printing is recognized as a versatile and competitive technology that can be classified into continuous inkjet printing (CIJ) and drop-on-demand (DOD) jetting according to the working principle [256]. The fabrication process involves ejection and photopolymerization of tiny photo-curable droplets on flexible substrates [45]. In CIJ, inks are propelled continuously from a reservoir and emitted through a nozzle in a non-contact manner and at high speed. As the name implies, DOD only expels droplets by thermal or piezoelectric effect from the nozzle when required [257]. The advantages of inkjet printing include high resolution, material efficiency, and on-demand multi-material customization [258–260]. However, the lack of suitable inks still hindered its industrial applications. Ongoing advances in the introduction of nanomaterials for the modification of inks have been proven effective in expanding the capabilities of tissue engineering, bioprinting, and wearable electronics [261–263].

Unlike traditional inkjet printing, AJP is a novel contactless and maskless approach in the field of microelectronic fabrication [264]. Notably, the AJP system is applicable to handle a wide range of raw inks for fine microstructures [265]. This complex technique employs an atomized stream and an inert carrier gas to precisely deposit inks in the form of focused and directed aerosol sprays onto various build platforms [266,267]. Furthermore, the integration of dispersed nanomaterials remarkably enhances and tailors the conductivity and mechanical properties of AJP inks [268–271]. These exceptional benefits attract ample interest in AJP for electronics and sensing areas [272–274].

As discussed above, each 3D printing technique comes with its strengths and limitations. Table 5 provides a concise summary of the characteristics of discussed methods, highlighting their principles, advantages, limitations, and application fields relevant to 3D-printed wearable devices [183].

## Application of nanomaterials in 3D-printed wearable devices

Notably, the unique properties of nanomaterials have effectively augmented the sensitivity and accuracy of sensing elements. Additionally, the advancement of 3D printing technology also revolutionizes the fabrication approach for wearable devices. The incorporation of nanomaterials with 3D printing has triggered the revolutionary development of more sophisticated and multifunctional wearable devices for health monitoring. Based on their applications, these 3D-printed and nanomaterial-enhanced wearable devices can be broadly classified into three main categories: physiological parameter monitoring (e.g., strain sensors and pressure sensors), biomedical signal monitoring (e.g., electrocardiogram (ECG) sensors and electroencephalography (EEG) sensors), and metabolic status monitoring (e.g., biosensors, and pH sensors). Table 6 summarizes the 3D-printed wearable sensors discussed in this review.

### Physiological parameter monitoring

Physiological parameters, such as physical pressure, blood pressure, pulse and other indicators, are essential for monitoring the body’s movement and detecting early signs of physical health issues, reflecting overall physical condition. Examples of sensors in this category include strain sensors and pressure sensors [275,276].

**FIG. 7:**
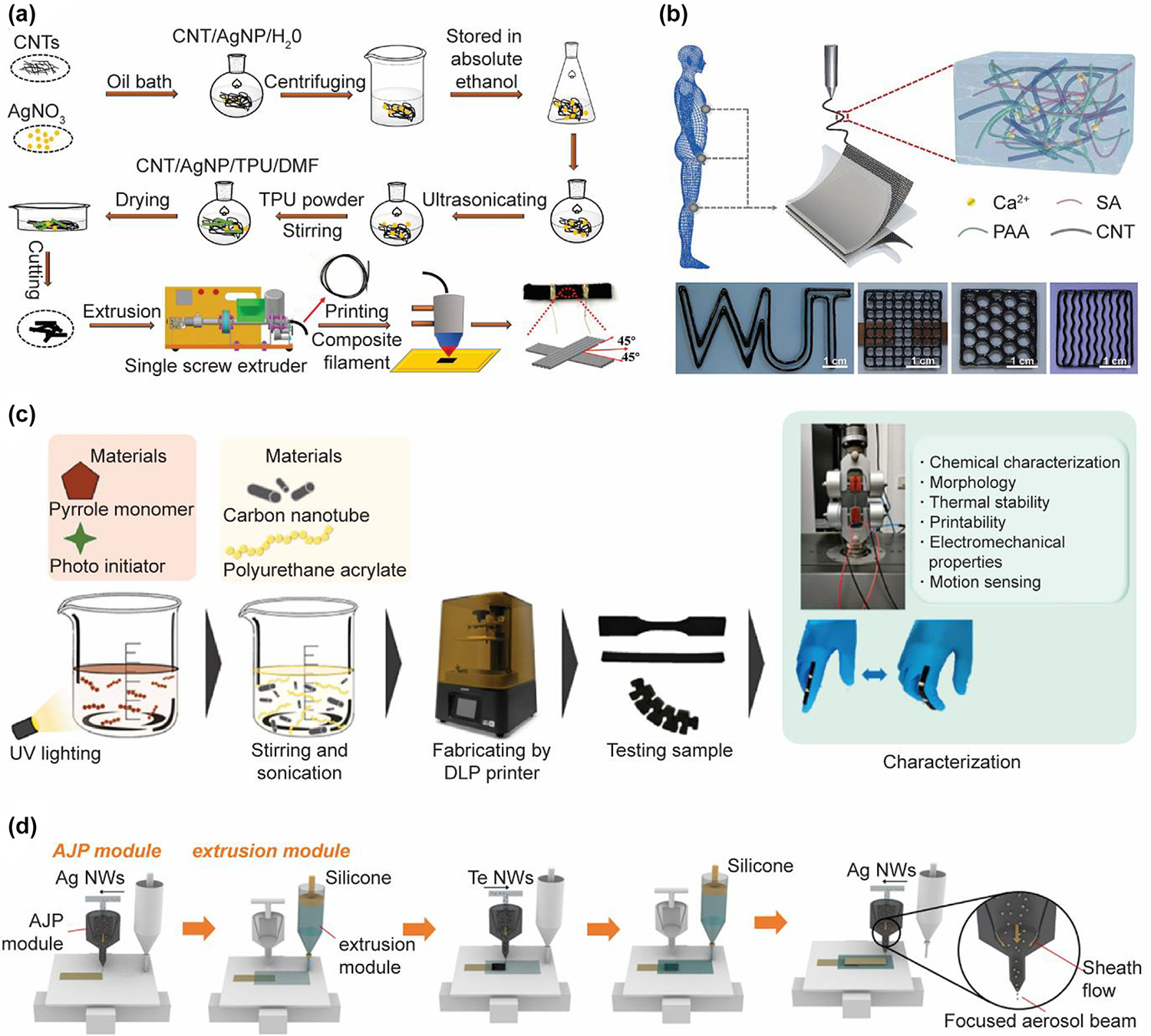
3D-printed strain sensors. (A) FDM 3D-printed strain sensor based on ternary CNT/AgNP/TPU composites. Reproduced with permission from Ref. [278]. Copyright 2020 Elsevier; (B) Extrusion-based 3D-printed and Ca-PAA-SA-CNTs hydrogel-based strain sensor Reproduced with permission from Ref. [279]; (C) Fabrication process of FPU/CNT/PPy composites for DLP printed strain sensors. Reproduced with permission from Ref. [280]. Copyright 2021 American Chemical Society [279]; (D) Hybrid printing process of the piezoelectric sensors using the aerosol jet printing and extrusion-based printing methods. Reproduced with permission from Ref. [281]. Copyright 2021 Elsevier.

Wearable strain sensors are capable of converting deformation change into a variation in electrical resistance, which have been widely used for the inspection of human joint motion. Nanomaterials are typically added to form conductive networks as active sensing materials to incorporate with stretchable supporting materials to fabricate wearable strain sensors [277]. Xiang et al. [278] reported 3D printed highly elastic strain sensors (Fig. 7A). CNTs and AgNPs were added into TPU to prepare ternary CNT/AgNP/TPU composites as the filaments for FDM printing. The synergistic effects of hybrid conductive nanofillers improved the printability, sensing and electrical properties of the filaments and rendered the sensors high sensitivity and linearity, fast response, outstanding stability, and repeatability for human motion monitoring. Wei et al. [279] fabricated skin-like wearable strain sensors with bioinspired hydrogels (Fig. 7B). Multiple conductive capabilities of their hydrogel were achieved by incorporating CNTs into a chelate of calcium ions with polyacrylic acid and sodium alginate. The hydrogel was self-healable, stretchable, and electronically and ionically conductive. After extrusion-based 3D printing, the integrated strain sensors exhibited excellent sensitivity and stability to detect subtle pressure changes caused by minimal movements and recognize the written signatures. Poompiew et al. [280] demonstrated DLP 3D-printed wearable strain sensors from flexible and electrically conductive polyurethane/CNT/PPy (FPU/CNT/PPy) resin composites with high complexity and model specifications (Fig. 7C). The prepared strain sensors were highly sensitive to response to small human motions. [279] Du et al. [281] presented a tellurium nanowire-based piezoelectric sensor fabricated by integrating AJP and extrusion printing methods. The formed nanowire network of tellurium nanowires and AgNWs eliminated the need for post-processing and featured sufficient stretchability and conductivity. The hybrid 3D-printed piezoelectric sensors efficiently detect the gesture and pulse, providing a novel strategy for the fabrication of wearable devices (Fig. 7D).

**FIG. 8:**
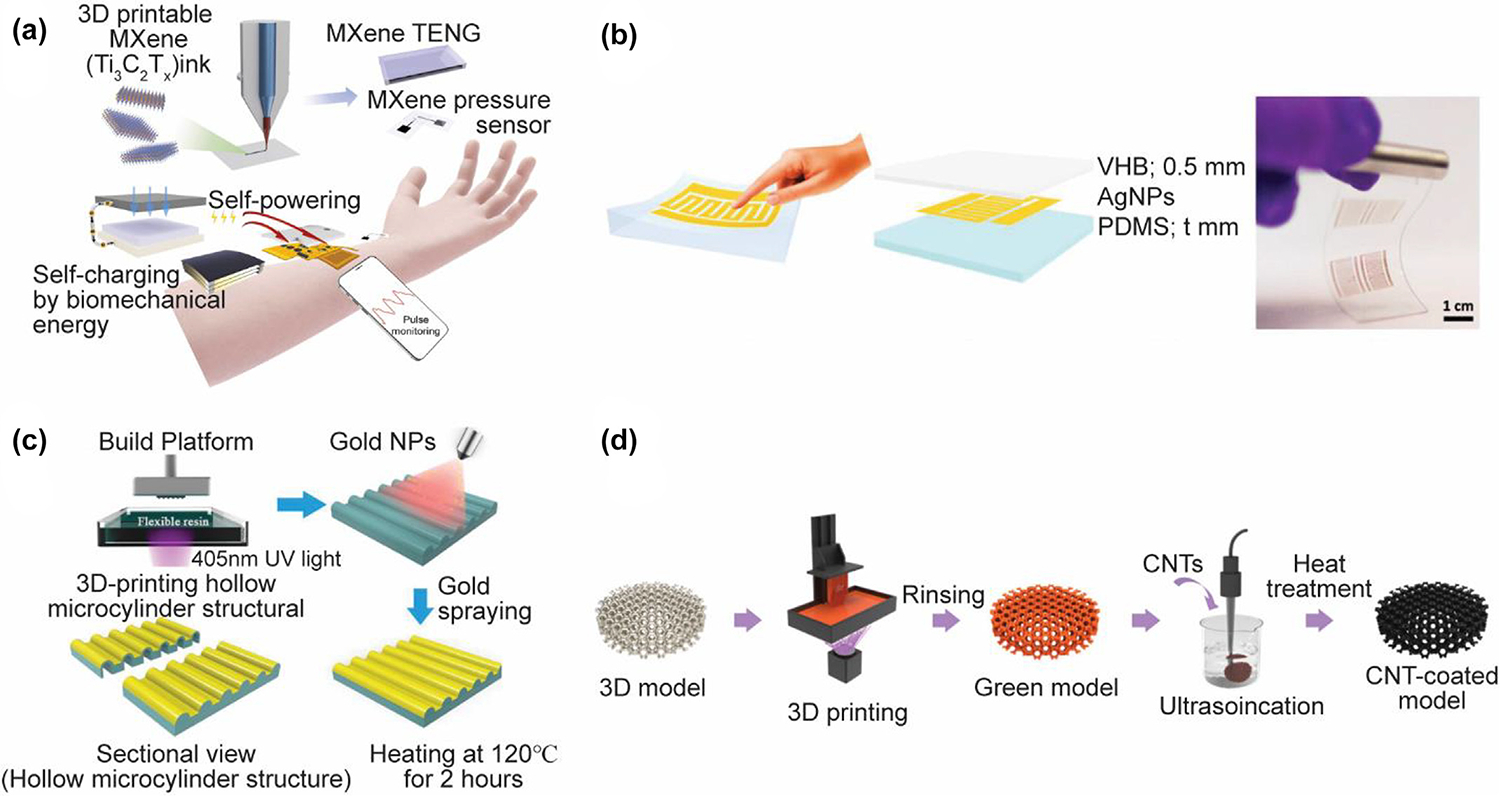
3D-printed pressure sensors. (A) Schematic of the DIW printed MXene-based sensing system. Reproduced with permission from Ref. [286]. Copyright 2022 Elsevier; (B) Working principle of the inkjet-printed resistive pressure sensor. Reproduced with permission from Ref. [287]. Copyright 2019 WILEY-VCH; (C) Fabrication process of flexible pressure sensors with 3D-printed hollow microstructures by an SLA printer. Reproduced with permission from Ref. [120]. 2021 Wiley-VCH GmbH; (D) DLP fabrication process of the flexible pressure sensor with a designable lattice structure. Reproduced with permission from Ref. [288]. Copyright 2021 American Chemical Society.

Wearable pressure sensors attract tremendous attention for electronic skin, touch interface, and wearables applications [282–284]. Nanostructured sensing materials ensure high sensitivity and stability for monitoring pressure signals [285]. Yi et al. [286] developed a 3D-printed and self-powered triboelectric MXene-based wearable sensing system (Fig. 8A). Ti_3_C_2_T_x_ MXene ink was extruded on the styrene-ethylene-butylene-styrene substrate. The integrated system was capable of continuous, wireless, and on-demand physiological sensing without the need for any extra power supply. Luo et al. [287] demonstrated an inkjet-printed resistive pressure sensor. The silver nanoparticles were patterned on an elastomer PDMS substrate and sandwiched by another VHB tape. Pressure-induced microcracks inside the AgNPs thin film resulted in the vibration of the resistance, making this sensor promising for smart wearable devices (Fig. 8B). Xia et al. [120] fabricated flexible pressure sensors with ultrahigh sensitivity for electronic skins. As shown in Fig. 8C, the hollow microcylinder structure was fully printed by the SLA method in one simple step, followed by the spray-coating of AuNPs. The assembled sensor was able to detect human throat swallowing and artery pulse signals. Yin et al. [288] introduced a DLP-printed flexible pressure sensor with a designable lattice structure (Fig. 8D). The embedded CNTs and subsequent thermal curing treatment ensured high electrical sensitivities and ultra-low detection limit of their sensor for human voice recognition, human pulse and foot pressure monitoring.

### Biomedical signal monitoring

**FIG. 9:**
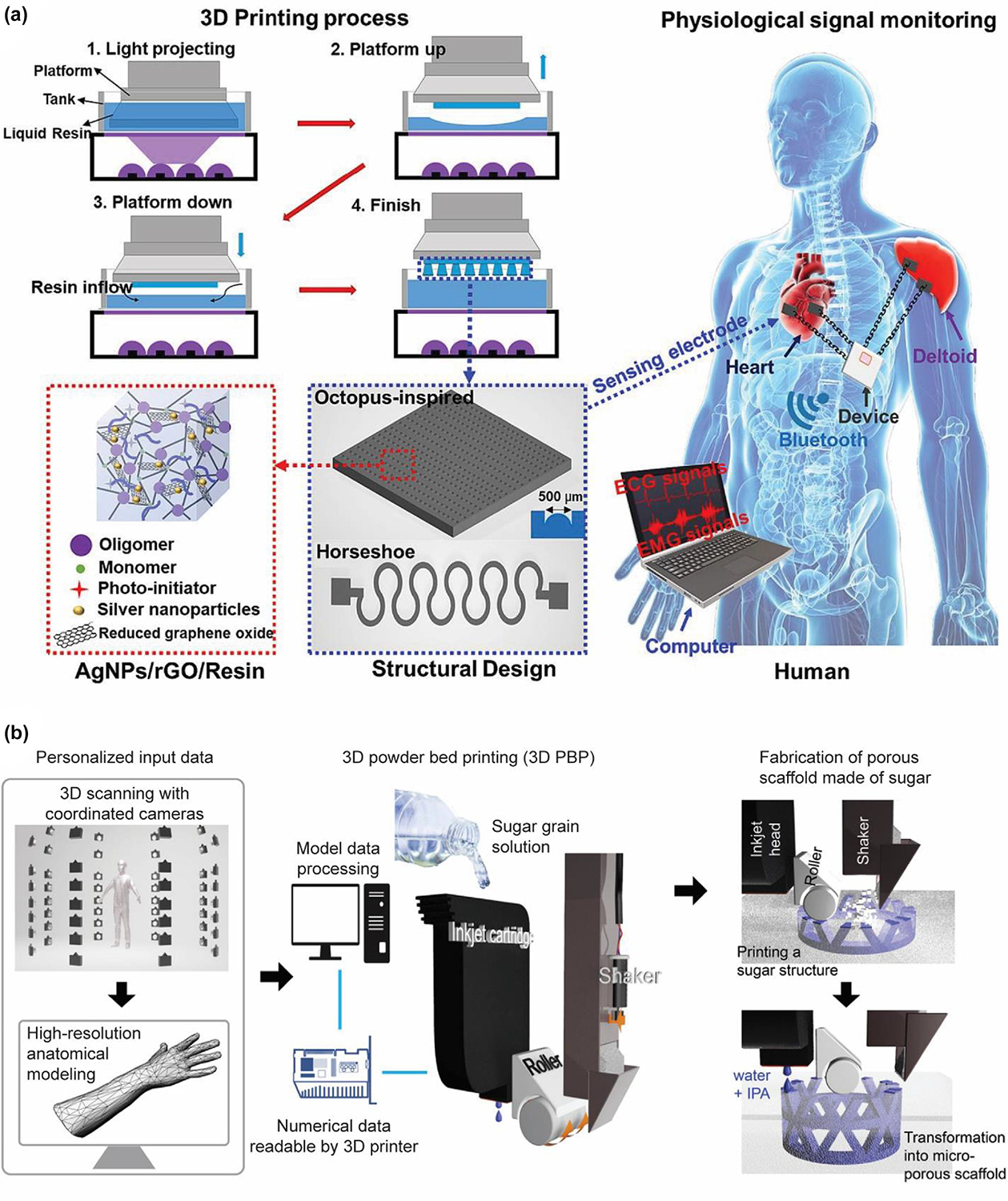
3D-printed ECG/EEG sensors. (A) DLP-printed sensing electrodes with octopus-inspired structure using graphene/elastic photocurable resins were attached to the skin near the forearm for ECG monitoring. Reproduced with permission from Ref. [292]. Copyright 2024 Elsevier; (B) 3D printing process via powder bed printing and printable porous materials for the hierarchical patient-specific wearable devices. Reproduced with permission from Ref. [293].

Noninvasive and wearable ECG and EEG sensors are transforming the way to monitor the bioelectric signals of the heart and brain activities, respectively [289]. However, the amplitudes of these signals are within the microvolt range [290]. Moreover, their instability and low signal-to-noise ratio require corresponding sensors with higher sensitivity [291]. 3D printing is becoming one of the most state-of-the-art technologies for electrode manufacturing. As shown in Fig. 9A, Li et al. [292] presented highly conductive flexible electrodes by the DLP method. A highly conductive photocured AgNPs/graphene/nanocomposite resin was modified and exploited to print stretchable electrodes for ECG measurement during exercise. Ho et al. [293] introduced a novel 3D printing technique and soft materials to fabricate patient-specific wearable devices to measure both actively and passively changing bio signals, including body strain signals and EEG, electromyography as well as electrodermal activity. The 3D-printed porous and flexible scaffold was coated with single-walled CNTs for electrical conductivity. The fabricated sensors successfully measured the bio signals for personalized point-of-care applications (Fig. 9B).

### Metabolic status monitoring

**FIG. 10:**
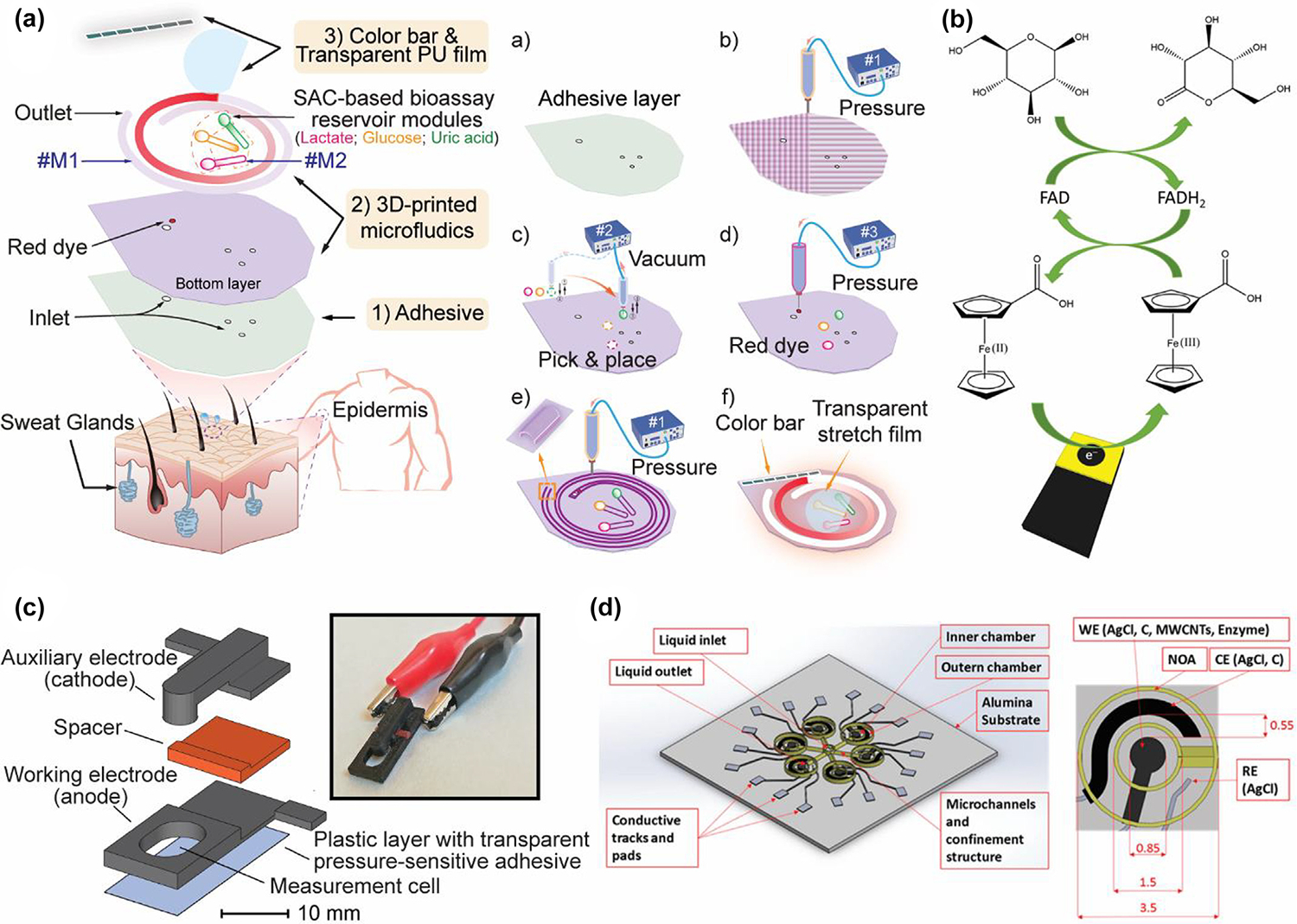
3D-printed biosensors. (A) Schematic of the DIW-printed flexible microfluidic health monitor and fabrication process from (a) place adhesive layer to (f) final prototype. Reproduced with permission from Ref. [43]. Copyright 2024 American Chemical Society; (B) Schematic of the GOx biosensor by FDM. Reproduced with permission from Ref. [88]. Copyright 2019 Elsevier; (C) FDM-printed electrochemiluminescence device. (a) Design and (b) image of the 3D-printed ECL device, (c) design and (d) image of 3D-printed dark box for measurement. Reproduced with permission from Ref. [165]; (D) Design of microfluidic sensors platform fabricated by AJP. Reproduced with permission from Ref. [296].

Metabolic conditions can be evaluated by tracking changes in biomarkers or electrolytes in body fluids, offering crucial insights for the management of related diseases and metabolic disorders. Examples of sensors include biosensors for the detection of specific biomarkers, and pH sensors for identifying metabolic balances [294,295]. Wearable biosensors provide physiological insights into human health monitoring and disease diagnosis, as shown in Fig. 10. Chen et al. [43] presented a flexible wearable health monitor with self-supporting microfluidic channels and novel single-atom catalyst (SAC)-based bioassays by direct ink writing and pick-and-place strategy in one continuous step (Fig. 10A). Nanomaterials enhanced the sensitivity and selectivity for the quantitative detection of glucose, lactate, and uric acid. Their proof-of-concept work validated the integration of the 3D printing method and nanomaterials enabled noninvasive monitoring of sweat component concentrations. Cardoso et al. [88] demonstrated a graphene-polylactic acid FDM printed platform for the analysis of biological fluids. They applied mechanical polishing and solvent treatment to improve the electrochemical performance. The fabricated platform can simultaneously evaluate glucose in blood plasma, nitrite and uric acid in urine and saliva, respectively (Fig. 10B). Calabria et al. [165] proposed an electrochemiluminescence biosensor for glucose monitoring (Fig. 10C). They used both conductive and non-conductive polymers to fabricate components via the FDM method. This point-of-care platform offered easy and reproducible solutions for real-time analytical systems. Novo et al. [296] reported an easy-of-use electrochemical sensor with microfluidics by aerosol jet printing (Fig. 10D). AJP microfluidic printing eliminated the need for supporting materials and multi-wall CNT ink was chosen to elevate and functionalize the electrical performance of the working electrodes. The high-resolution platform was able to quantify glucose with good replicability and sensitivity.

**FIG. 11:**
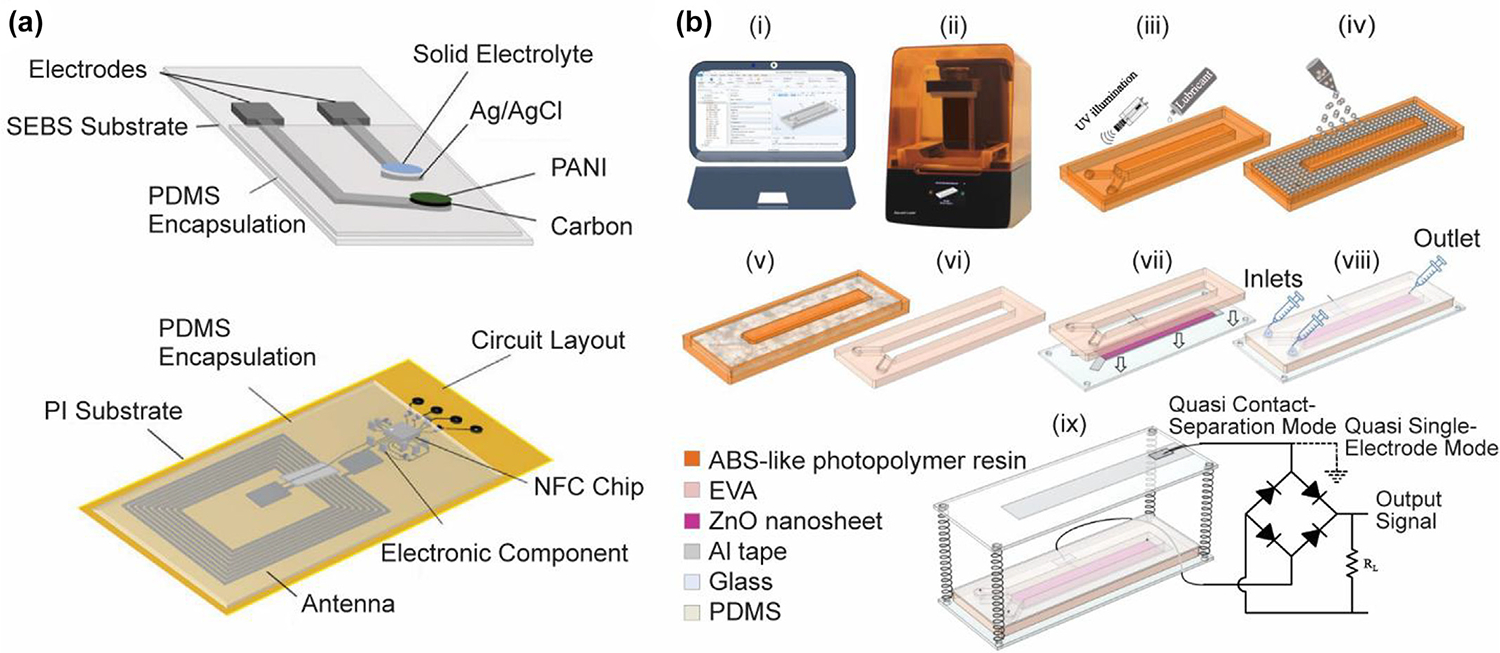
3D-printed pH sensors. (A) Schematics of DIW-printed WB^2^F3D sensor for on-demand and real-time pH sensing. Reproduced with permission from Ref. [300]. Copyright 2023 Wiley-VCH GmbH; (B) schematic of DLP fabrication process of the millifluidic-based pH sensing device from (i) CAD design to (ix) setup of the sensor. Reproduced with permission from Ref. [301]. Copyright 2023 Elsevier.

pH sensors are designed to monitor the pH levels of bodily fluids, which is crucial for diagnosing chronic skin lesions and managing the infection process [297–299]. NajafiKhoshnoo et al. [300] fabricated a 3D-printed multi-functional wearable sensor system for real-time pH monitoring (Fig. 11A). Silver nanoparticles were used to print electrodes and encapsulated for biocompatibility. The integrated WB^2^F3D sensor provided dynamic and *ex situ* pH monitoring. Ghofouri et al. [301] developed a simple and rapid 3D-printed millifluidic device (Fig. 11B). Their proof-of-concept work exhibited high sensitivity and versatility for sweat pH sensing.

## Outlook

With its extensive customization capabilities, 3D printing can effectively meet the practical requirements of wearable devices, particularly in terms of lightness and flexibility [303]. Meanwhile, excellent properties, such as high stretchability, ultra-flexibility, low cost, ultra-thin and lightweight, make 3D-printed wearable biological devices become the next-generation tool for healthcare applications [37]. However, the incorporation of nanomaterials into 3D-printed devices can endow various devices with customized mechanical, chemical and electrical functionalities [304]. Nanomaterials can provide various unique properties and functions, including mechanical properties, flexibility, electrical conductivity, and biocompatibility, to the 3D-printed wearable monitoring devices [183,305,306]. The integration of nanomaterials is driving 3D printing technology into a new stage in wearable monitoring devices. On the contrary, 3D printing provides an effective platform in improving stability and biocompatibility and enhancing the dispersibility of nanomaterials [51].

Although nanomaterials usually improve printing resolution and surface finish, the nanoscale size also brings problems to the 3D printing [271]. For example, particles in the size range of 0–150 μm tend to aggregate due to strong van der Waals forces, regardless of the presence of liquid [307]. For polymer nanocomposites used in 3D printing, direct mixing of polymers and nanofillers can cause aggregation of nanomaterials. However, nanomaterials synthesized *in situ* allow effectively prevent aggregation and precipitation of nanomaterials and are well distributed in the polymer network [308]. Notably, AgNPs can be synthesized *in situ* and used to fabricate polymer/AgNP nanocomposites [309,310]. Meanwhile, a suitable combination approach for dispersing nanomaterials into matrix materials prior to the printing process is also a feasible solution [90,311]. Two approaches named *ex situ* and *in situ* are commonly used to stabilize the nanomaterials against their aggregation when dispersing nanomaterials in polymer matrix. These approaches require the formation of a polymer coating on the nanomaterials to enhance biocompatibility and make the nanomaterials more easily dispersed within the polymer matrix. In the *ex situ* process, the polymer shell is made by external force to obtain nanomaterials uniformly dispersed in the polymer system. However, *insitu* process mainly relies on chemical interactions between reactive functional groups (such as van der Waals interactions, hydrogen bonds, etc.) to achieve crystal growth based on carbon allotropes or polymerization of other materials [312]. This strategy also solves the poor correlation between nanomaterials and matrix materials.

The nanoscale size also makes them more likely to diffuse in the air, affecting the reliability of the printer’s components [271]. In addition, nanomaterials exhibit high surface area to volume ratio and high reactivity [313]. The presence of oxygen may affect the properties of the material which needs strict requirements in the 3D printing environment. In this case, a printer equipped with an atmospheric control system is required to provide an inert printing environment [271].

The safety of nanomaterials used in the fabrication of wearable monitoring devices is critical to their applications. Their nanoscale size allows them to potentially penetrate and diffuse within the body. From this respect, it is essential to incorporate biocompatible nanomaterials, such as biomaterials, into the development of safe, bio-friendly and sustainable 3D-printed wearable monitoring devices [42,314,315]. Biomaterials with excellent biocompatibility are used in bioprinting to fabricate biological structures that replicate living tissue [183]. The most common materials for bioprinting are gels that mimic the extracellular matrix, which promotes cell colonization [316,317]. The development of biomaterials and the application of bioprinting provide the possibility for developing scaffold-based or scaffold-free tissues or organs and replicating patient tissues [318].

State-of-the-art artificial intelligence and machine learning (AI/ML) are revolutionizing the design and optimization of 3D-printed wearable devices by enabling predictions of device performance, and optimizing nanomaterials, enhancing sensing accuracy [319,320]. For example, ML plays a crucial role in optimizing or classifying the performance of wearable devices formed with various nanomaterials, including carbon dots [321], graphene [322], silver nanowires [323], AuNWs [324], nanocomposites [325], silver fibers [326], polymer fibers [327] for specific applications. Adaptive algorithms improve wearable sensors for motion monitoring, such as gait analysis [328], while ML techniques are applied to detect biomarkers like lactate [329] or to monitor mental health [330] using 3D-printed wearable devices. Furthermore, deep neural networks and generative algorithms hold significant potential for designing advanced wearable monitoring systems, offering real-time health tracking with high accuracy.

## Summary

The incorporation of nanomaterials and 3D printing technologies has revolutionized the performance and manufacturing process of wearable devices, though it is still in its nascent stage. The obstacles, including nanomaterial functionalization, materials limitations for fabrication, 3D printing constraints, and integration approaches for nanomaterial-enhanced 3D-printed wearable devices, remain challenging. This article comprehensively highlights the unique characteristics of nanomaterials including CNTs, metal nanoparticles, MOF, and biomaterials, which enhance multiple properties for sensitive and precise health monitoring. Additionally, the working principles, advantages, and limitations of 3D printing techniques, particularly, materials extrusion, vat photopolymerization, and material jetting, are extensively reviewed. The synergy between nanomaterials and additive manufacturing has driven remarkable progress in various wearable devices for physiological monitoring and biochemical sensing. We envision that, through interdisciplinary collaboration and joint effort, next-generation wearable devices hold great promise for advancing human healthcare.

## Figures and Tables

**TABLE 1:** 3D-printed wearable monitoring devices based on carbon nanomaterials.

Materials	Methods	Advantages	Applications	Ref.
Graphene	SLA	Outstanding electrical conductivity, high sensitivity, cost-effectivity, efficient sense	Microneedle biosensor for real-time ketone and glucose monitoring.	[75]
Graphene/PLA	FDM	Electrochemical properties, high detection precision	Electrode for bioanalysis of glucose, uric acid and nitrite simultaneously.	[88]
CNT-silicone composite	Direct ink writing (DIW)	Flexible, stretchable, printing without prior mixing, curing under ambient conditions	Wearable electronics for motion detection, cardiac and respirator monitoring devices, and bionic skin application.	[92]
CB	FDM	Electrochemical property, cheap, fast responses, suitable analytical performance	Electrochemical platform for adrenaline detection.	[93]
CNFs	Two-photon nanolithography	High sensitivity to multiple neurochemicals, high reproducibility, robust enough for tissue implantation	Microelectrodes to detect dopamine.	[94]

**TABLE 2:** 3D-printed wearable monitoring devices based on metal nanomaterials.

Materials	Approaches	Functions	Applications	Ref.
AuNPs	AJP/IJP	Low-cost; low-temperature sintering; high resolution; high-quality print lines	For wearable sensors fabrication using AJP or IJP methods	[107]
AuNPs	SLA	Excellent stability; good compression performance; rapid response and recovery	For human physiological signals monitoring and electronic skins	[120]
AuNPs-MXene	IJP	Ultra-low limit of detection; amplification-free; real-time analysis	Wearable sensors for personalized monitoring of female hormones (oestradiol)	[108]
AuNPs	DIW	Rapid response and recovery; stability; wide resistance range	Wearable and flexible humidity-sensing devices	[121]
AuNW	Photolithography	Reproducibility, stability; fast response	Noninvasive wearable sweat analysis of pH, Na^+^, and K^+^ in sweat	[122]
AgNPs	AJP	High conductive, biocompatible	High-performance, capacitance flow sensor for monitoring cerebral aneurysm hemodynamics	[113]
AgNPs	DIW	Sustainable and biocompatible	Fabrication of ion selective membrane electrodes for 3D-printed disposable wireless ion sensors	[123]
AgNPs/PLA	FDM	Highly monodisperse; good surface hydrophilicity	Cell culture scaffolds; biosensors; wound healing devices	[124]
AgNPs	IJP	Continuous monitoring, smartphone-based readout	Wearable biosensor for Cu and heavy metals in sweat.	[125]
AgNPs	DIW	Good repeatability and stability; excellent recovery rate	Fabrication of complex-structured electrodes for determination of H_2_O_2_	[114]
Cu/Sn/Zn NPs/GnP	DIW	Good flexibility and durability; oxidation resistance	Strain sensor	[126]
Copper nanoparticles	FDM	Robustness, low cost	3D-printed Cu electrodes for the non-enzymatic sensing of glucose	[127]
Cu/PLA composite	FDM	Eliminate kinetic barrier	Patterned Cu-modified 3D-printed electrodes for electrochemical sensor	[128]
Fe (III)	FDM	Low cost; enzyme free	Integrated 3D-printed wearable biodevices for glucose detection	[129]

**TABLE 3:** 3D-printed wearable monitoring devices based on MOF.

Nanomaterials	Techniques	Functions	Applications	Ref.
Fe (II)-MOF	FDM	Low cost, non-enzyme, stability, non-toxicity	Sensor for glucose sensing in human sweat	[142]
Zr-MOF (MOF-525)	FDM	Mechanical strength.	colorimetric sensor for monitoring various human body movements	[144]
ZIF-8	DIW	Peroxidase activity, stability, low cost, sensitivity, accuracy	Flexible microfluidic health monitor for *in situ* sweat analysis	[43]
Eu-MOF	SLA	Excellent efficiency; outstanding reproducibility; excellent selectivity; excellent biocompatibility	Minimally invasive skin-worn device for detecting cortisol in ISF	[151]

**TABLE 4:** 3D-printed wearable monitoring devices based on biomaterials.

Nanomaterials	Technologies	Functions	Applications	Ref.
Shear-thinning hydrogels	DIW	Compressive modulus, biocompatibility	3D-printed flexible biosensors, actuators, and therapeutic delivery devices	[160]
poly(HEMA) hydrogels	Digital light processing (DLP)	Strong adhesion, detachable	Contact lens for colorimetric pH and electrochemical Na^+^ sensing	[161]
Agarose hydrogel	FDM	Simple fabrication, fast response, excellent selectivity and sensitivity	Smartphone-based 3D-printed electrochemiluminescence enzyme biosensor for glucose quantification	[165]
P(SBMA-co-AAc)/CS-Cit DN hydrogel	DIW	Stretchable, anti-fatigue, self-healing properties	3D-printed wearable strain sensor for detecting human motions	[166]
PDL/AgNWs	DIW	Antimicrobial, biocompatible properties	Flexible strain-responsive sensor	[167]

**TABLE 5:** Characteristics of 3D printing methods for wearable devices.

3D Printing methods		Principle	Advantages	Limitations	Relevant application fields
Material extrusion	Fused deposition modeling (FDM)	Filament extrusion	Simple, low cost, multi-material	Limited resolution, require post-processing and support structures	Wearable sensors, biomedical engineering, electronics, soft actuators and robotics
	Direct ink writing (DIW)	Pressurized extrusion of viscoelastic ink	Versatile	Low resolution, nozzle clogging	
Vat photopolymerization	Stereolithography (SLA)	UV polymerization	High resolution, excellent surface finish	Limited resins, require post-processing	Tissue engineering, biomedical engineering, photonics, and microfluidic devices
	Digital light processing (DLP)	Digital light projector for curing	High speed and resolution	Require post-curing and support removal	
	Two-photon polymerization (TPP)	Two-photon absorption and polymerization	Extremely high resolution	Expensive	
Material jetting	Inkjet printing (IJP)	Continuous inkjet, Drop-on-demand	High throughput	Complex process	Wearable sensors, biomedical engineering, bioprinting, and
	Aerosol jet printing (AJP)	Aerosolized droplets delivered by inert carrier gas	High resolution	Material limitations, complex	electronics

**TABLE 6:** Summary and characteristics of 3D-printed wearable sensors discussed in the article.

Sensor		Material	3D printing method	Application	Ref.
Physiological parameter monitoring	Strain sensor	CNT/GNP/TPU	FDM	Limb motions, physiological activities, and speech recognition	[278]
		Ca-PAA-SA-CNTs hydrogels	DIW	Bioinspired materials in the field of flexible smart devices	[279]
		FPU/CNT/PPy	DLP	Human motion detection	[280]
		TeNW/AgNW	AJP	Wearable and biomedical sensors	[281]
	Pressure sensor	Mxene/SEBS	DIW	Continuous and real-time physiological biosignals monitoring	[286]
		AuNPs/resin/PET/Ag	SLA	Human physiological signals such as cheek bulging, throat swallowing, and artery pulse	[287]
		CNTs/TPU	DLP	human pulse, voice signal recognition and foot pressure	[302]
		AgNPs/PDMS	IJP	Disposable wearable sensor	[288]
Biomedical signal monitoring	ECG/EEG sensor	AgNPs/graphene/polymer nanocomposites	DLP	ECG and EMG smart clothing	[292]
		SWCNT-network-coated silicone elastomer	3D PBP	EMG, EDA, and EEG	[293]
Metabolic status monitoring	Biosensor	Fe-N-C SACs	DIW	Sweat rate, glucose, lactate and uric acid	[43]
		graphene-PLA	FDM	Biological fluids	[88]
		Conductive and conventional PLA, glucose oxidase	FDM	Glucose in pharmaceutical and biological samples	[165]
		MWCNT/Ag/C/AgCl	AJP	Electrochemical microfluidic sensing platform	[296]
	pH sensor	AgNPs/SEBS/PDMS	DIW	Real-time human health monitoring	[300]
		ZnO nanosheet/resin	DLP	Millifluidic pH sensor	[301]

## Data Availability

Data will be made available on request.
